# Grating-Coupled Plasmonic Resonances in Symmetric 2D Gold Nanobump Grating: Theory Meets Experiments

**DOI:** 10.1021/acsami.6c04043

**Published:** 2026-05-19

**Authors:** Kernius Vilkevičius, Lucciano A. Letelier, Lina Grinevičiu̅tė, Evaldas Stankevičius

**Affiliations:** † Plasmonics and Nanophotonics Laboratory, Department of Laser Technologies, Center for Physical Sciences and Technology (FTMC), Savanoriu Ave. 231, LT-02300 Vilnius, Lithuania; ‡ Optical Coatings Laboratory, Department of Laser Technologies, Center for Physical Sciences and Technology (FTMC), Savanoriu Ave. 231, LT-02300 Vilnius, Lithuania

**Keywords:** grating-coupled resonance, plasmonics, periodic gold nanostructures, femtosecond laser, direct laser writing, FDTD simulations

## Abstract

The excitation of hybridized plasmonic modes is induced by periodic, two-dimensional gratings. Hybrid lattice plasmon resonances (HLPRs) emerge from the interaction of surface plasmon polaritons (SPPs), localized surface plasmons (LSPs), and grating-induced light diffraction and scattering. These resonances are characterized by dispersive and narrow line widths, which are particularly relevant for biosensing and surface-enhanced spectroscopies. In this comprehensive investigation, the diffraction-based excitation of HLPR resonances in a symmetric 2D gold nanobump grating is examined with excitation occurring across multiple lattice planes. The resonance tunability with respect to azimuthal and incidence angles, as well as polarization, is experimentally investigated. To identify and validate the origins of the resonant peaks, theoretical calculations of the spectral and near-field responses were conducted, providing a thorough examination of the phenomena. This combined theoretical-experimental study highlights the pivotal role of symmetry and excitation geometry in defining hybrid plasmonic modes, thereby providing guidelines for the engineering of highly tunable plasmonic platforms.

## Graphical abstract

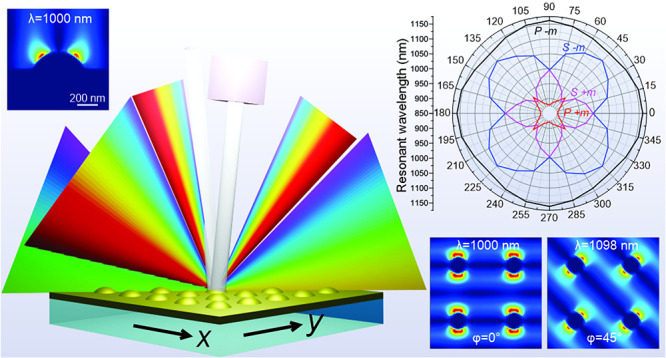

## 1 Introduction

Periodic metallic nanostructures support a variety of plasmonic resonances arising from the interaction of fundamental plasmonic modes and incident radiation. One of these hybrid modes arises from the coupling between repetitive localized surface plasmons (LSPs) and diffraction-induced propagating surface plasmon polaritons (SPPs).^1–3^ The hybridization of plasmons is obtained in the periodic nanostructure array when the wave is diffracted at the cutoff angle, emanating from the metal surface, and the Rayleigh anomaly condition is fulfilled.^4^ When the localized surface plasmons of neighboring individual nanostructures and the scattered field are in phase, plasmonic oscillations and local electromagnetic field are enhanced, resulting in reduced radiative losses and efficient far-field coupling. This interaction between repetitive LSPs and a diffracted ray gives rise to a hybrid mode known as surface lattice resonance (SLR), whose key features are a narrow line width and strong spectral tunability.^5–7^ The addition of a gold layer among the periodic nanostructures leads to the excitation of the SPP by the same in-plane diffracted ray at the metal-dielectric interface.^8^ Consequently, the coupling of LSP-SPP modes is initiated, and a hybrid lattice plasmon resonance (HLPR) is obtained. Although SPPs can be excited by the presence of a glass prism, the excitation of this HLPR mode is achieved exclusively with a diffraction grating.^9^ In two-dimensional gratings, the coexistence of multiple diffraction planes enables the simultaneous excitation of plasmonic modes along orthogonal symmetry axes and diagonals.

The resonant properties of these hybrid plasmonic systems strongly depend on the structural grating parameters and are tunable by tailoring the period and symmetry of the grating,^10–14^ as well as the particle geometry and dimensions,^15,16^ and the number and arrangement of nanostructures in the array.^17–20^ The results are also influenced by sample parameters such as incidence angle and polarization,^21–23^ the plasmonic metal, the adhesion layer, and the surrounding medium.^15,24,25^ To ensure stable resonant properties, the nanostructures must be uniform in shape, and the metal surface must be chemically stable (nonoxidizing). Hybrid resonance phenomena are widely used for accurate, powerful sensing of biomolecules, protein binding, analyte solutions,^26–28^ tumors,^29^ and gases,^30^ which depend strongly on changes in the surrounding refractive index. The use of resonant structures is also a method of enhancing the Raman scattering signal in the vicinity of nanostructures.^31–34^ Plasmonic lattices and hybrid modes have also been investigated for nonlinear optical response and lasing,^35–37^ fluorescence,^38^ and used for boosting other energy conversions,^39^ as well as for fluid flow manipulation,^40^ nonradiative cooling,^41^ and anticounterfeit labels.^42^

While periodic metallic nanostructures can be manufactured in several ways,^43–45^ a single-step large-scale direct laser writing (DLW) technique was implemented in this fabrication. Ultrafast laser processing of the sample, by ablating or modifying the metal layer, enables accurate microprocessing at the focal point of the laser beam.^46–48^ By applying a single laser pulse to modify the layer, energy-dependent nanostructures can be obtained from the heated and melted metal layer. Previous studies have shown that the formation of these structures depends strongly on the laser wavelength and pulse energy,^49^ the metal used,^50^ and its thickness.^32,51^ The plasmonic properties of such nanostructures also depend on these parameters, and the most prominent dependence is morphology, as the resonant wavelength redshifts with increasing height and morphological phase of the structure.

The diffraction-based plasmonic excitation can be described using either a fully complex 2D or a simplified 1D periodic-array model. In the literature, the latter 1D case is more commonly investigated because the phenomena are reduced to a single axis, allowing clearer analytical treatment and simpler interpretation of the underlying physics.^52–54^ As a result, many plasmonic concepts are typically introduced and analyzed within a 1D framework. However, a 2D grating description is essential when studying systems with hybrid-mode formation, multiaxis diffraction, and multidirectional plasmonic coupling. Symmetric arrays have been widely investigated in the context of SLR excitation and transitional conditions between individual LSP and SLR modes.^55–57^ Additionally, grating-coupled SPP mode excitation in two-dimensional lattices under orthogonal incidence conditions, as well as the Bragg mode excitation in hybrid plasmonic-photonic crystal systems, have been explored for practical implementations.^27,58^ Despite extensive studies and analysis on 1D gratings and geometries, the full angular dependence of plasmonic resonances in symmetric 2D lattices, particularly the interplay between main-axis and diagonal diffraction orders, remains insufficiently explored. This study aims to experimentally and theoretically investigate grating-coupled plasmonic resonances in symmetric 2D gold hemispherical nanobump gratings fabricated on thin films. By systematically varying polarization, azimuthal orientation, and incidence angle, we resolve the dispersion and hybridization behavior of multiple diffraction-based resonances. Numerical simulations are used to model both the spectral response and near-field distributions, enabling a clear classification of the observed resonances to their origins. This combined experimental-theoretical approach clarifies how diffraction order, propagation direction, and polarization jointly shape the hybrid plasmonic response in 2D gratings.

## 2 Results and Discussion

### 2.1 Grating-Coupled Resonances in 2D Gold Nanobump Arrays

Large-scale periodic symmetrical 2D arrays of gold nanostructures on a 100 nm thin film were fabricated using the DLW technique, when the third-harmonic (343 nm) pulses of 300 fs Yb:KGW-based laser Pharos (Light Conversion Ltd.) were utilized in this study. The focused beam size was 0.8 μm, achieved with a high numerical aperture (NA) objective of NA = 0.5. The fabrication process entailed scanning periodic lines at a constant sample translation speed and pulse repetition rate (7 kHz) to structurize thin metal films and obtain nanostructures with a period of 1 μm in the scanning lines. In order to produce a symmetric 2D grating, the same period of 1 μm was used between the scanning lines. The structures were produced using a 0.8 nJ single-pulse energy to obtain hemispherical bumps.

The fabricated periodic nanobump arrays excite a hybrid plasmonic mode that propagates along the metal surface. The SPP mode propagation wavevector at the metal-dielectric interface is described as follows:^59^

$$k_{\mathrm{SPP}}=\frac{2\pi }{\lambda }\sqrt{\frac{\varepsilon_{\mathrm{Au}}}{1+\varepsilon_{\mathrm{Au}}}}$$ 1

where ε_Au_ is the permittivity of the gold film. The SPP excitation is based on light diffraction from the grating and Wood’s anomaly, when the specific wavelength of the beam diffracts at a grazing angle so that it propagates along the surface (Figure 1a). The periodic grating changes the momentum of the incident light, leading to a matching of photon and SPP wavevectors and a consequent SPP excitation. Nanobump array changes the incident light wavevector *k*_*i*_ = 2π/λ by an integer number of grating wavevectors *k*_g_ = 2π/*s*, where *s* is the grating period; thus, the resonant wavelength highly depends on the distance between the structures in both axes. For SPP excitation in a 2D array in an air environment, the following diffracted wave condition must be met:^60^

$$\begin{array}{c} k_{\mathrm{SPP}}=k_{i}\sin\theta \pm mk_{g,x}\pm nk_{g,y} \end{array}$$ 2

where θ is the angle of incidence, *k*_g*,x*_ and *k*_g*,y*_ correspond to grating wavevectors in the *x* and *y* axes of the array, which, in this case of a symmetrical square grating, are identical, and *m* and *n* are integers representing diffraction orders in *x* and *y* directions. This includes an additional resonant-wavelength control via azimuthal and incidence angles. Therefore, the coupling between a surface plasmon and a diffracted wave is fulfilled when eqs 1 and 2 are matched and can be achieved by different diffraction orders. In addition to the SPP, since the nanostructures are of close proximity to the light wavelength in size, the LSPs are excited on each of the periodic structures. Therefore, the hybridization of LSPs, SPP, and the diffracted ray at a grazing angle occurs in the symmetric 2D gold nanobump grating. During the measurements, the grating period was kept constant, with only polarization, the angle of incidence θ (angle between the incident beam and the surface normal), and the azimuthal angle φ (angle between the plane of incidence and the laser scanning axis) being investigated (Figure 1b).

**Figure 1:**
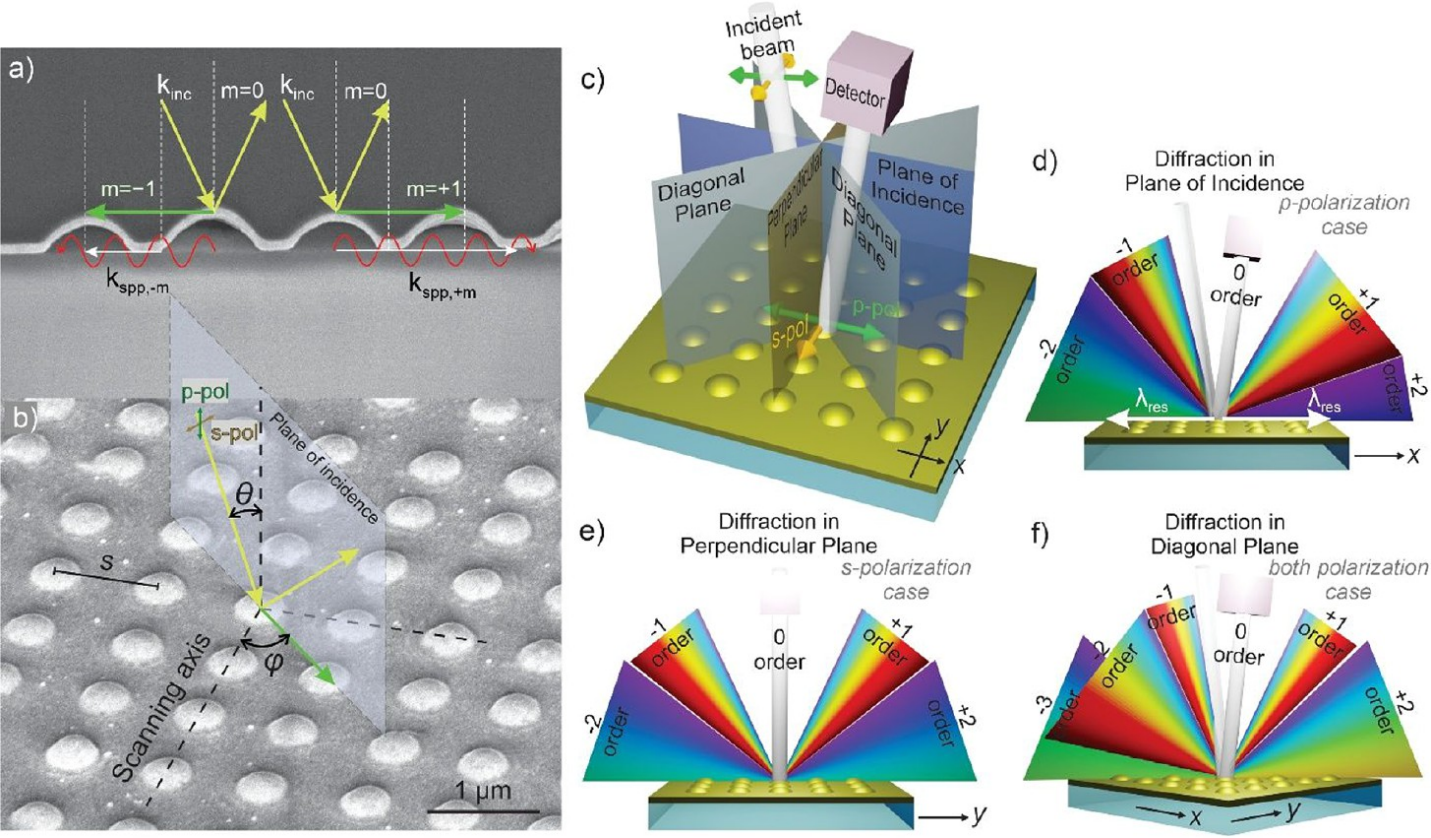
(a) Cross-section of the bumps with grating-coupled excitation of surface plasmons. (b) SEM micrograph of gold bumps grating with marked plane of incidence and angles used in measurements. (c) 3D drawing of the main diffraction planes at an 8° incident angle beam at the fabricated gold nanostructures grating. Diffraction in (d) plane of incidence (PI), (e) perpendicular plane (PP), and (f) diagonal plane (DP). The spectral visualization of diffraction is depicted for the wavelength range of 400–800 nm. White arrow marks resonant wavelength.

The beam impinging to the crossed 2D grating exhibits a conical diffraction, having the four main planes of diffraction (Figure 1c): Plane of incidence (PI, Figure 1d), that is the plane of the incident and the reflected beam, perpendicular plane (PP, Figure 1e), that is perpendicular to the plane of incidence, and 2 diagonal planes (DP, Figure 1f), that are at 45° with both PI and PP. The positive and negative diffraction orders are different in PI and DP (*m* = +1 and −1, *m* = +2 and −2), while in PP, these are similar (*n* = ±1, ±2) due to the symmetrical diffraction conditions. The p-polarized light has an electric field lying in the PI, while the s-polarized light has an electric field parallel to PP. As mentioned, plasmons are excited when the beam is diffracted at 90° to the normal and is parallel to the surface (the cutoff wavelength). Thus, in such a grating of 1 μm period, the beam diffracted in the first order excites them with the IR spectral part, while the second-order diffracted beam, with a visible or UV range wavelength.

The plasmonic response of the fabricated periodic gratings was measured using a spectrophotometer (Photon RT, Essentoptics). The reflectance spectra of the arrays were analyzed to determine the position of the plasmon resonance, as evidenced by a decrease in reflectance upon absorption of the incident radiation. A light beam with a 2 mm-diameter circular spot was used. The measurements were obtained using both s- and p-polarized radiation, in the spectral range of 400–1600 nm with a step of 4 nm. Most measurements were performed at a single angle of incidence (θ = 8°); however, to assess the effect of angle, measurements were also obtained at 15° and 30°. Additionally, the azimuthal angle φ of the sample was changed by 15° so that φ = 0° was considered when the spectrophotometer plane of incidence was parallel to the laser scanning lines, φ = 45° when oblique to the scanning lines, and φ = 90° perpendicular to them.

Since the presented DLW method enables easy morphological tuning by adjusting the deposited energy, it is possible to produce differently shaped structures within the same grating in a single fabrication step. This allows the production of distinct profiles between adjacent or in each second line in the grating. Spectral comparison of uniform bumps, bump-cone, and bump-jet gratings showed that the sharpest resonant peaks are obtained with bump gratings, and greater morphological variation leads to broader, less-pronounced resonances. This can be attributed to reduced effective resonance coupling and wider height variations for the out-of-plane resonant mode. Although such more complex, mixed-morphology structures are of broader interest, the analysis of grating-coupled excitation described in this work is most clearly distinguished in uniform-bump gratings, which exhibit the sharpest and most distinct resonances.

Figure 2a,b shows the measured reflection spectra for s- and p-polarizations at azimuthal angles φ = 0°, φ = 45°, and φ = 90° for the bumps grating with a 1 μm period. In the spectra, at φ = 0° (Figure 2a,b; black line) and φ = 90° (Figure 2a,b; blue line), the first-order diffraction resonance behavior is similar for both polarizations: one resonance in the PP at s-polarization (996 nm), and two resonances in the PI at p-polarization (1162 and 877 nm). In addition to these usual first-order 1D-origin diffraction resonances, other diffraction peaks are observable. To identify their origin, spectra were measured by rotating the sample around its azimuthal axis. The near-identical spectra at φ = 0° and φ = 90° imply that the grating is uniform along both the scanning and perpendicular axes. Meanwhile, the φ = 45° spectra (Figure 2a,b; red line) prove the plasmon excitation not only in the main periodic array of 1 μm period, but also in a diagonal lattice of √2 μm effective period. Also, the 2D crossed gratings support multiple diffraction periodicities defined by the in-plane lattice vectors (*k*_*x*_, *k*_*y*_). The 1D-related resonances are associated with (1,0) and (0,1) lattice vectors and are excited in PI and PP, while those of 2D origin, with (1,1) or higher-order lattice vectors, are excited in DP. In further analysis, the observed peaks will be separated into two groups: primary resonances (denoted as *E*_pr_), excited at longer wavelengths in the 850–1200 nm range, and secondary resonances (*E*_se_), excited at shorter wavelengths in the 600–850 nm range. Near the edge of absorption (550–600 nm range), shallow tertiary resonances may also be observed but will not be widely investigated.

**Figure 2:**
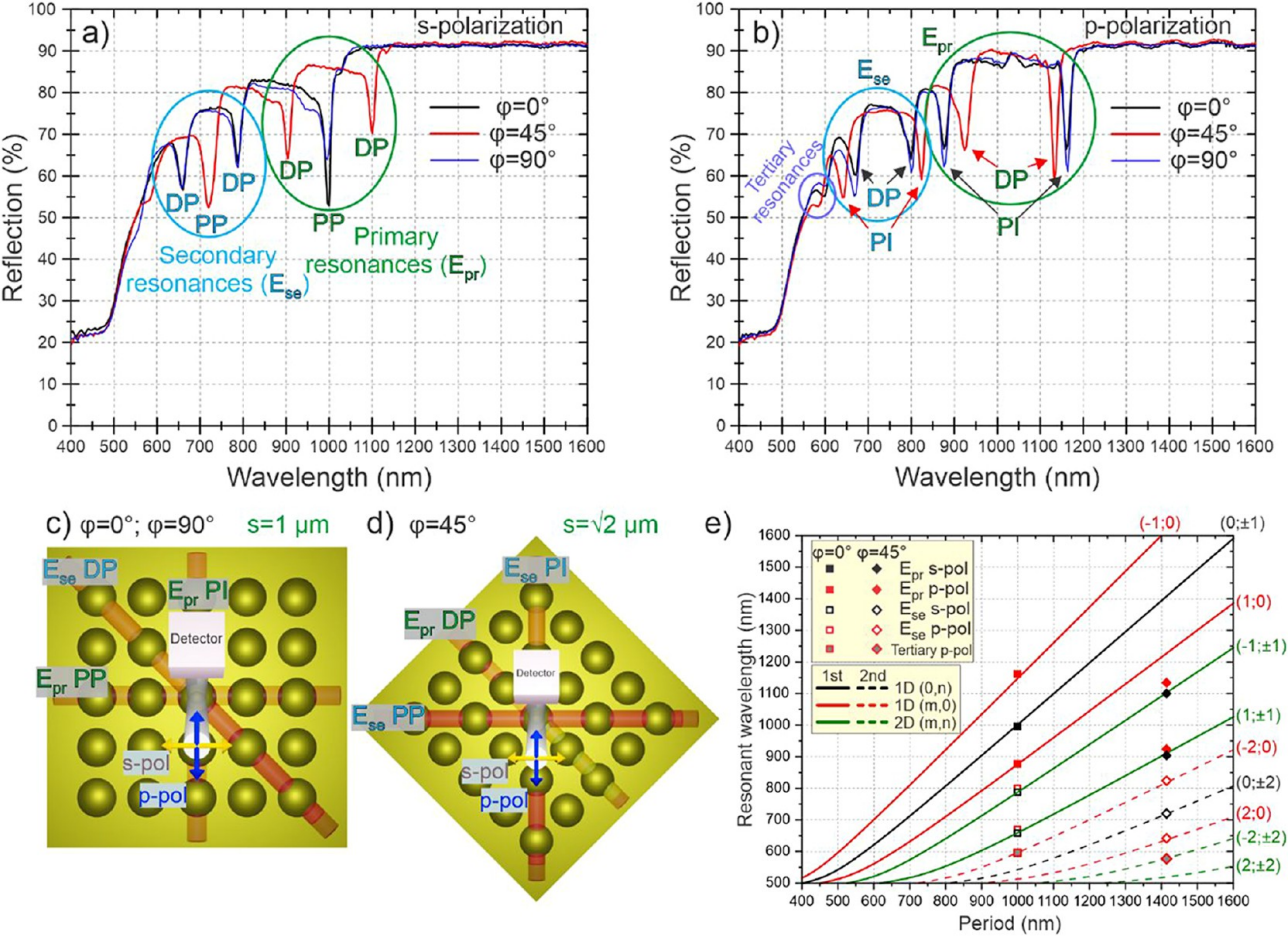
Reflectance spectra of gold nanobumps grating with a period of 1 μm for (a) s- and (b) p-polarization. The sample was measured at three azimuthal angles φ = 0° (black line), φ = 45° (red line), and φ = 90° (blue line). The marked primary resonances (*E*_pr_, green) and secondary resonances (*E*_se_, cyan), originating in the plane of incidence (PI), perpendicular plane (PP), and diagonal plane (DP) at (c) φ = 0°/90° and (d) φ = 45°. (e) Theoretically calculated resonant wavelength dependence on the grating period at an 8° incident angle for 1D-origin s- (black lines) and p-polarization (red lines), as well as 2D-origin (green lines). The first diffraction order is depicted in solid lines, while the second order is in dashed lines. The symbols mark experimentally measured resonance values from (a) and (b) graphs. Primary (filled symbols), secondary (empty symbols), and tertiary (gray symbols) resonances are marked by squares (φ = 0°) and diamonds (φ = 45°) for s- (black) and p-polarization (red).

For s-polarization (Figure 2a), at φ = 0°/90°, two secondary *E*_se_ dips are observed below the single primary peak *E*_pr_ of the first-order diffraction at PP. These originate from the 2D-type diffraction in DP (788 nm for negative and 658 nm for positive orders). The resonances in the measured spectra at φ = 45° change as the single 1D-type *E*_pr_ resonance originating at PP splits into two (positive and negative) 2D-type resonances, which are excited in the DP of the rotated sample (1100 and 904 nm). Meanwhile, the two secondary *E*_se_ peaks observed from the DP at φ = 0° merge into a single peak (720 nm) at φ = 45°, as it is now excited at symmetrical PP. The transition from two peaks at φ = 0° into one at φ = 45° confirms that this secondary resonance in PP is of 1D-type diffraction origin from a diagonal grating.

In p-polarization spectra (Figure 2b), the same resonance origins are observed, with the difference that instead of a single resonance from PP, the two resonances from PI appear. Meanwhile, the resonances originating in DP (*E*_se_ at φ = 0° and *E*_pr_ at φ = 45°) occur at rather similar spectral positions in both polarizations (Figure S1), with minor shifts attributed to alignment inaccuracies at φ = 0° and φ = 45° angles, leading to the coupling in a diagonal plane and 2-dimensional diffraction that is less sensitive to polarization. The two *E*_pr_ 1D-type resonances at φ = 0°/90° originating from PI (1162 and 877 nm) shift closer to each other (1134 and 924 nm) as the azimuthal angle is changed to φ = 45°, and *E*_pr_ are then excited in DP. At the shorter-wavelength region, the *E*_se_ are observable, where the ones originating in PI at φ = 45° are in a wider wavelength range (824 and 642 nm) than the ones excited in DP at φ = 0°/90° (800 and 670 nm). Finally, the tertiary resonances are observed in the lowest wavelengths (596 nm at φ = 0°/90°) just before the gold absorption zone. All the components that are visible in Figure 2a,b are visualized in Figure 2c,d with respective directions.

Since the resonant excitation is of diffractional origin, the diffraction grating equation can be employed to theoretically evaluate and compare the experimentally observed resonant wavelengths. For a squared grating, the 2D diffraction formula for plasmonic excitation can be applied to calculate the resonant wavelength:^61^

$$\begin{array}{c} \lambda_{\mathrm{res},2D}=\frac{s}{\left(m^{2}+n^{2}\right)}\left(-m\sin\theta \pm \sqrt{\frac{\varepsilon_{\mathrm{Au}}}{1+\varepsilon_{\mathrm{Au}}}(m^{2}+n^{2})-n^{2}\sin^{2}\theta }\right) \end{array}$$ 3

where *s* is the grating period, *m* is the diffraction order in direction parallel to the plane of incidence, *n* is the diffraction order perpendicular to the plane of incidence, θ is the angle of incidence, and ε_Au_ is the dielectric constant of gold. According to this notation, the diffraction of 2D-origin is denoted as (*m;n*).

As the crossed 2D grating is composed of two orthogonal 1D arrays, the theoretical values can also be calculated using the simplified 1D diffraction grating formulas,^52,62^ which represent special cases of the 2D formula. When the electric field oscillations are perpendicular to the 1D grating lines (diffraction in PI, p-polarization case), the grating vector is parallel to PI, while the perpendicular component does not contribute. In this (*m*;0) case, the resonant wavelength is expressed as

$$\begin{array}{c} \lambda_{\mathrm{res},1D,p-\mathrm{pol}}=-\frac{s}{m}\left(\sin\theta \mp \sqrt{\frac{\varepsilon_{\mathrm{Au}}}{1+\varepsilon_{\mathrm{Au}}}}\right) \end{array}$$ 4

For the case when the electric field oscillations are parallel to the grating lines (diffraction in PP, s-polarization) and PI does not contribute, the (0*;n*) case wavelength is given by

$$\lambda_{\mathrm{res},1D,s-\mathrm{pol}}=\pm \frac{s}{n}\sqrt{\frac{\varepsilon_{\mathrm{Au}}}{1+\varepsilon_{\mathrm{Au}}}-\sin^{2}\theta }$$ 5

The calculated resonant wavelength dependence on the grating period at a constant incidence angle of θ = 8° is presented in Figure 2e. The results are shown for the first (solid lines) and second (dashed lines) orders of 2D-type (green lines) and 1D-type (black and red lines) diffraction. Black lines correspond to s-polarization (0;*n*), while red lines correspond to p-polarization (*m;*0). 2D-type diffraction is observed at lower wavelengths than one-dimensional diffraction, occurring between the first and second orders. The experimental data extracted from the spectra in Figure 2a,b are indicated by discrete symbols: data points at a period of 1 μm for φ = 0°/90° are shown as squares, while those at a √2 μm period for φ = 45° are shown as diamonds. Filled symbols denote the primary resonances (*E*_pr_), whereas empty symbols correspond to secondary resonances (*E*_se_), and filled gray symbols mark the tertiary resonant wavelengths. Great agreement between the experimental data and the theoretical curves enables unambiguous identification and assignment of the resonances to their diffraction origins.

At φ = 0°, the observed *E*_pr_ resonances are attributed to the first-order 1D-origin diffraction. In s-polarization, the resonance in PP corresponds to the (0; ±1) diffraction, and in p-polarization, the resonances in PI are (−1;0) and (1;0). Meanwhile, the *E*_se_ of both polarizations excited at DP are associated with the first-order 2D-type diffraction. Specifically, the resonances near 800 nm are assigned to (−1; ±1), and the ones around 650 nm correspond to (1; ±1). Under p-polarization excitation, a shallow tertiary resonance is observed below 600 nm. It coincides with the (−2;0) curve, indicating that higher-order modes of both repetitive 1D-type and 2D-type diffractions are excited at shorter wavelengths. At φ = 45°, the excitation occurs in a diagonal grating with √2 μm effective period. Here, primary resonances *E*_pr_ for both polarizations are excited in DP and accurately match the 2D-origin diffraction curves of (−1; ±1) and (1; ±1). S-polarization data (black solid diamonds) fit perfectly with the theoretical line, while the p-polarization results (red solid diamonds) exhibit a slight deviation. This discrepancy can be attributed to the oblique excitation and the presence of an out-of-plane component in the p-polarized field, whereas the s-polarized field remains entirely in-plane. Secondary *E*_se_ resonances (empty diamonds) coincide with second-order 1D-type diffraction curves: p-polarized resonances excited in PI correspond to (−2;0) and (2;0), and s-polarization in PP matches the (0; ±2) curve. Again, under p-polarized light, observed tertiary resonance below 600 nm matches the (−2; ±2) curve, further confirming the repetitive excitation of higher-order modes. In this diagonal case, the first-order 1D-type diffraction at longer wavelengths is not observable, meaning the efficiency of this component is too low for the excitation. Summarizing the observations, azimuthal rotation alters the lattice of excitation, leading to a changed resonance origin. Main grating (φ = 0°/90°) primarily excites 1D-type diffractional resonances in PI and PP, with the following 2D-type diffraction peaks obtained in DP. In a diagonal grating (φ = 45°), primary resonances originate from 2D-type diffraction in DP, followed by higher-order 1D-type diffraction in PI and PP. This whole analysis and attribution of the peak origin is summarized in Figure S2.

The obtained plasmonic peaks were characterized by measuring their width and depth and by determining the quality factor (Q-factor), defined as the ratio of the resonant wavelength to the resonance width (*Q* = λ/fwhm). Also, a modified quality factor (MQ-factor) including the depth of the peak *h* was calculated for the comparison of its intensity:

$$\begin{array}{c} \mathrm{MQ}=\frac{\lambda \cdot h}{\mathrm{FWHM}\cdot 100\%} \end{array}$$ 6

The exact evaluation of the peak depth and MQ-factor is described in.^50^ The *E*_pr_ resonance qualities were measured at azimuthal angles of φ = 0°, φ = 45°, and φ = 90° to compare the values. The results are given in Table 1. The calculated values confirm a previously determined trend:^62^ resonance in s-polarization is stronger at φ = 0°, whereas in p-polarization it is stronger at φ = 90°. However, the difference in quality is minimal, and both azimuthal angles can be used for measurements. At φ = 45°, when the diagonal grating becomes dominant, and the origin is changed from 1D to 2D-type, the qualities of shifted *E*_pr_ resonances increase a bit for negative and decrease for other orders of diffraction. Meanwhile, for the *E*_se_ component (Table 2), the qualities are higher at φ = 45° for p-polarization peaks when the excitation is 1D-origin. Peaks in s-polarization, however, are of higher quality at φ = 0°, since the one at φ = 45° is broader. The Q-factor of the described periodic gold bump reaches up to 70–80 for primary resonances *E*_pr_, while it is reduced to around 60 for secondary *E*_se_ resonances. The Q-factor is influenced by the variations in the structure size, shape, and periodicity, as well as diffractive coupling into several lattices simultaneously, which can reduce the coupling efficiency, broaden the peaks, and reduce this parameter. In the literature, the described Q-factor of self-assembled lattices reaches up to 20,^10^ higher resonance qualities (over 300) are achieved using isolated periodic nanoparticles that excite SLR,^24^ while similar periodic structures with a gold layer in between reach around 70–140,^63^ depending on the structure size.

**Table 1:** Q-factor and MQ-Factor of the *E*_pr_ Resonances in the Bump Grating at *φ* = 0°, 45°, 90° Azimuthal Angles

	Q-factor		MQ-factor	
azimuthal angle	s-polarization *–m/+m*	p-polarization *–m/+m*	s-polarization *–m/+m*	p-polarization *–m/+m*
φ = 0° (1D-type)	76.2	68.8/48.6	19.2	14.2/6.9
φ = 45° (2D-type)	83.5/66.8	74.6/44.4	11.5/9.15	21.1/6.2
φ = 90° (1D-type)	68.8	73.4/49.5	8.1	19.5/8.9

**Table 2:** Q-factor and MQ-Factor of the *E*_se_ Resonances in the Bump Grating at *φ* = 0°, 45°, 90° Azimuthal Angles

	Q-factor		MQ-factor	
azimuthal angle	s-polarization *–m/+m*	p-polarization *–m/+m*	s-polarization *–m/+m*	p-polarization *–m/+m*
φ=0° (2D-type)	58.3/38	36.6/35.3	7/4.4	4.4/3.3
φ=45° (1D-type)	28.45	59.5/36.8	4.9	8.9/3.7
φ=90° (2D-type)	57.1/35	45.9/33.9	7.2/3.7	6.7/3.7

### 2.2 Rotational Plasmonic Properties in the Azimuthal Angle

To gain a deeper understanding of resonance spectral manipulation, reflection spectra were measured at azimuthal angles from φ = 0° to φ = 90° in 15° steps. By changing this angle at a constant θ = 8° angle of incidence, a clearer resonance shift from φ = 0° to φ = 45°, and back is observed, as represented in Figure 3a,b. Each curve exhibits several reflection minima corresponding to plasmonic resonances excited at different diffraction orders and planes. Here, the primary *E*_pr_ resonances in the near-IR (850–1200 nm range) are circled in blue, and the secondary *E*_se_ resonances (650–850 nm range) are circled in green. The exact resonant wavelength value tendencies are depicted in Figure 3c,d, with blue lines showing *E*_pr_ and green lines marking *E*_se_. Upon rotation, the *E*_pr_ resonances split into two, reaching maximum separation at φ = 45° in s-polarization and at φ = 0° in p-polarization, whereas the *E*_se_ resonances show the opposite behavior. This periodic splitting-merging with φ is a consequence of the conical diffraction behavior occurring in the crossed grating. The excitational component of the incident wavevector is projected differently onto various lattices of the sample as it is rotated, and the change between 1D-type and 2D-type diffraction is happening.

**Figure 3:**
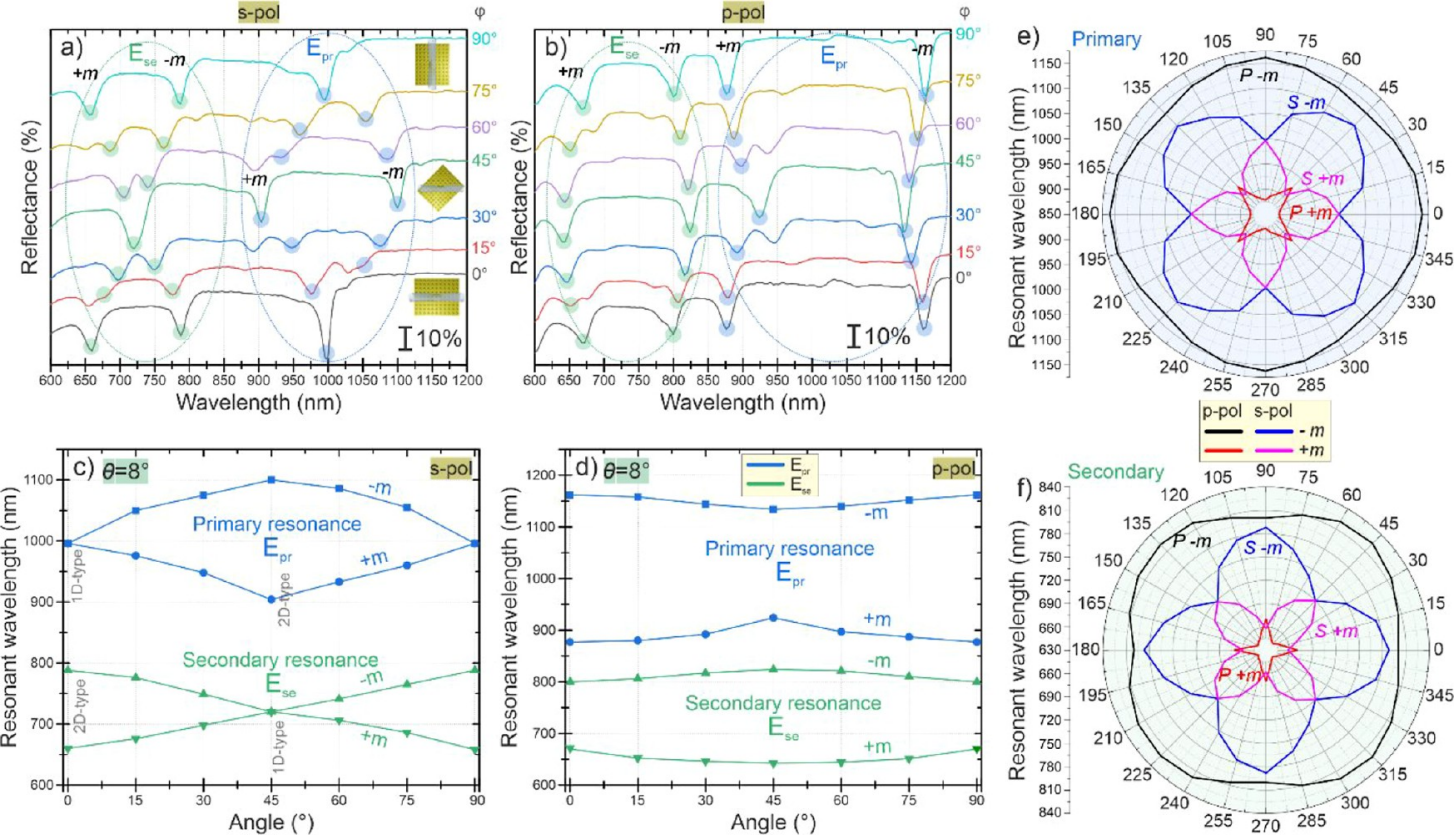
Reflectance spectra of gold nanobumps grating with a period of 1 μm at different azimuthal angles from φ = 0° to φ = 90° with a step of 15° for (a) s- and (b) p-polarization. Blue circles indicate the primary resonances, while green circles indicate the secondary resonances. Resonant wavelength shift with the change of azimuthal angle at a fixed θ = 8° angle of incidence for (c) s- and (d) p-polarization. Resonant wavelength change of (e) primary *E*_pr_ and (f) secondary *E*_se_ resonances in the polar coordinate system.

At the intermediate angles (15°, 30°, 60°, 75°), several shallow resonant peaks can be observed close to each other, with several of these attributed to the shifting resonance of either *E*_pr_ or *E*_se_ with the changed excitation and coupling lattice. At such angles, the incident wavevector is projected onto several lattices, and neither the main nor the diagonal grating diffraction condition is strictly satisfied. This leads to excitation coupling across several planes and to reduced coupling strength for each in-plane mode. For example, at φ = 15° in s-polarization, two shallow overlapping peaks are observed at 1050 and 1029 nm, which may arise from coupling to several lattices with similar effective periods. Also, some of the additional dips can be explained by the influence of another polarization, as denoted in Figure S3. At angles φ = 15° and φ = 75°, two *E*_se_ peaks at 652 and 678 nm, and at 651 and 684 nm, respectively, are observed in both polarizations, differing only in intensity. The same is observed for *E*_pr_ at φ = 30° and φ = 60°, the resonances being at 892 and 948 nm, and at 897 and 933 nm, respectively. Other resonant peaks are observed in both polarizations, albeit with lower intensities. Such a tendency illustrates the coupling between the two polarizations into main or diagonal gratings at nonperpendicular azimuthal angles. The depth and width of the resonant peaks are indicative of the coupling rate through the specific diffraction plane and of the metal’s intrinsic losses. Sharp and deep dips indicate efficient diffractive momentum matching, whereas broad, shallow features indicate weak coupling. The observed peak narrowing and broadening with φ indicate angle-dependent coupling, which was most effective for the (1,0) and (1,1) lattices at 0° and 45°, whereas at intermediate angles the peaks were shallow with modest diffractive coupling.

The polar plots in Figure 3e,f provide a visualization of the angular evolution of the resonant wavelength for the *E*_pr_ and *E*_se_ modes in the main and diagonal gratings. The resonances produce a four-lobed pattern aligned with either principal grating axis or along the diagonals, demonstrating a consistent pattern across the entire azimuthal rotation. The alignment of the lobes along the main axes demarcates the blueshifting peaks, while the lobes along the diagonals denote the redshifting resonances as the excitation plane undergoes a transition from main to diagonal grating (from φ = 0° to φ = 45°). A general trend emerges that is consistent across all resonances. The modes exhibit minimal splitting and merge upon excitation by 1D-type diffraction in PP. An intermediate interpeak distance is observed with 2D-type diffraction at DP, and the largest splitting is attained at 1D-type PI excitation.

To complement the experimental observations and verify the origin of the observed resonances, the optical response of the 2D periodic gold-bump grating was modeled using the Lumerical FDTD solver. To model the 3D structure (2D grating), we defined a unit cell with Bloch boundary conditions, based on Bloch–Floquet theory,^64,65^ in the direction perpendicular to the grating, and with perfectly matched layers (PMLs) in the light-propagation direction. This configuration enables the simulation of a periodic structure with a precise mesh. The unit cell, with a periodicity of 1 μm, consists of a dielectric material with refractive index *n* = 1.51 at 900 nm (Soda-Lime Glass^66^) and is topped by a 100 nm-thick gold layer. As illustrated in Figure 1a, the bump is modeled with a height of 270 nm and a diameter of 550 nm. The bump consists of a 65 nm-thick shell, and its interior is filled with a medium of refractive index *n* = 1. The gold material is described using the optical constants from Johnson and Christy.^67^ To account for the oblique incidence used in the experiment, the simulation employs Lumerical’s Broadband Fixed Angle Source Technique (BFAST), which enables oblique illumination by correcting the phase according to the incidence angle.^68^ The 3D simulation provides the transmission and reflection spectra and the electric-field distribution parallel to the surface, evaluated at different heights. In addition, the 3D model enables the extraction of the electric-field distribution along an oblique vertical cross-section, which cannot be obtained from standard two-dimensional simulations, by slicing the full 3D tensor field. Since the source is planar and normalized, the local *E*_pr_ and *E*_se_ fields represent an increment attributable to plasmonic resonances.

The simulated reflection spectra and near-field distributions provide direct insight into the diffraction-based plasmonic mode excitation and its dependence on polarization and azimuthal orientation. Figure 4 presents the spectra of s-polarized light, with black lines representing the simulated response and red lines representing the experimentally measured reflectance. Peaks marked in blue are attributed to E_pr_ resonances, while those marked in green are *E*_se_. A comparison of the simulated and experimental spectra and resonances reveals a strong coincidence in both spectral positions and overall peak behavior across the shifted azimuthal angle. There is only a slight shift of the simulated peaks toward longer wavelengths relative to the experimental peaks, which may arise from nonideal grating fabrication and the use of ideal hemispherical structures in the simulation. Consistent with the experimental observations, the strongest peaks, with the largest intensity drops, are observed at φ = 0° and φ = 45° for the *E*_pr_ resonances. Meanwhile, the peaks at intermediate azimuthal angles are low in intensity because the incident direction is not matched to the grating and is not optimally aligned with the dominant grating. Since the resonances at φ = 0° and φ = 45° are already identified, their origin is labeled, while for intermediate angles, peaks are not uniquely attributable to a single diffraction lattice and are therefore distinguished only by their positive (+*m*) and negative (−*m*) diffraction orders.

**Figure 4:**
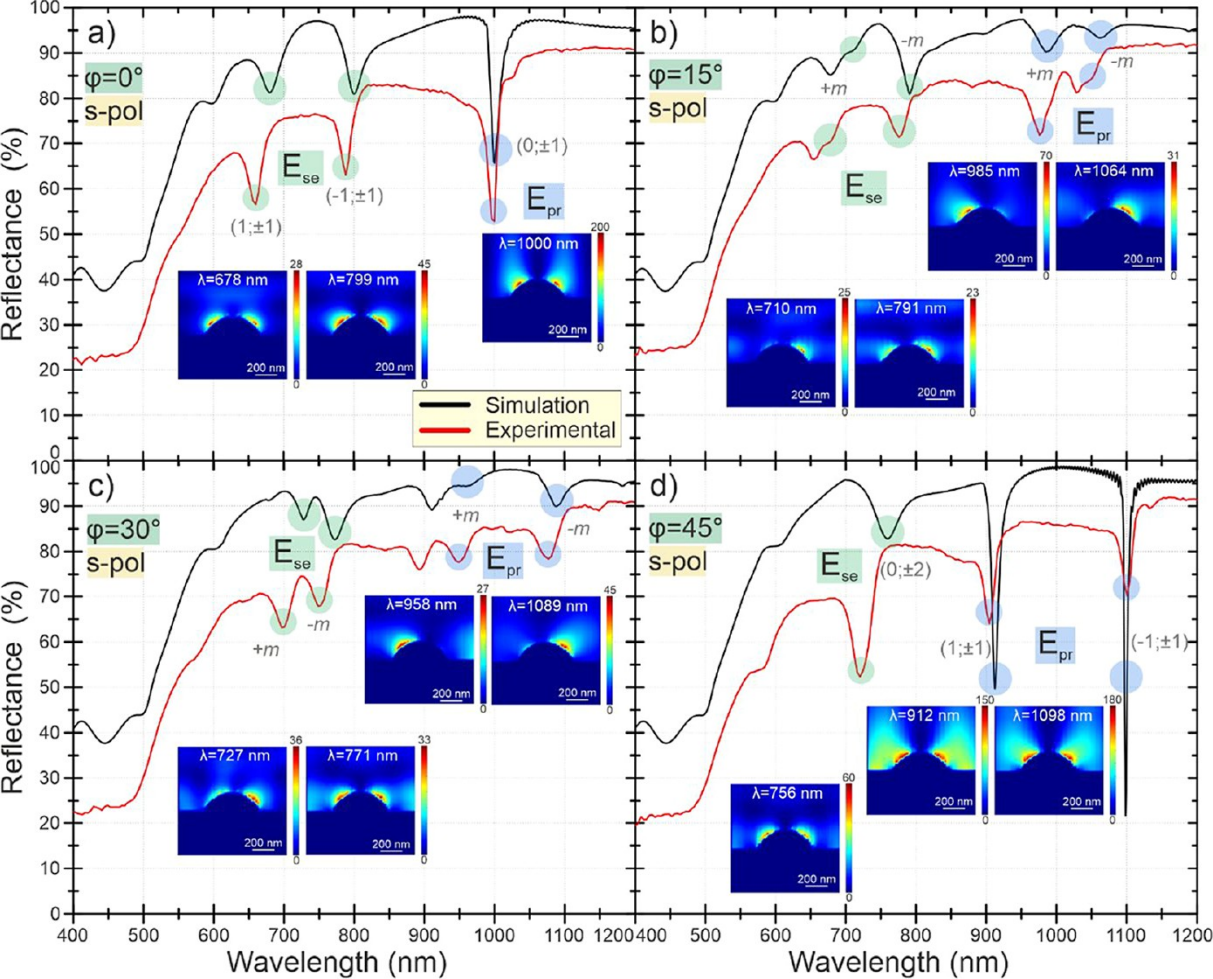
FDTD simulated (black line) and experimental (red line) reflectance spectra of s-polarized light response from gold nanobumps with a period of 1 μm at θ = 8°. The spectra are shown for azimuthal (a) φ = 0°, (b) φ = 15°, (c) φ = 30°, and (d) φ = 45°. Blue circles indicate the primary *E*_pr_ resonances, while green circles indicate the secondary *E*_se_ resonances. Insets show the side-view profiles of the simulated near-field enhancement on the structures at the corresponding resonant wavelengths.

The cross sections of the insets in Figure 4 are obtained at the plane of electric field oscillations according to the azimuth. All plasmonic excitations obtained with s-polarization are in-plane modes, with near-field enhancements along the sides of the structure. The field is distributed uniformly, with two lobes at angles where the incident plane aligns with the main or diagonal grating (Figure 4a,d), thereby enabling efficient coupling and deep resonances. Azimuthal changes to φ = 15° and φ = 30° result in asymmetric field localization on either side of the structure. This phenomenon may coincide with positive and negative diffraction orders, as observed for E_pr_ resonances, and appears due to the coupling not confined to the single lattice.

These simulations also enable the demonstration of the hybrid origin of the resonances (HLPRs) and their distinction from SLRs. Typical SLR systems are composed of isolated metallic nanostructures, and their LSPs are coupled via in-plane photonic diffraction modes, whereas the structures in this work are connected by a continuous gold film and coupled by surface waves (SPPs). Similar hybrid excitation in metallic structures with a thin film was observed in other publications.^1,69–71^ The simulation of the response spectra of our periodic bumps (HLPR excitation) and identical structures without the film (theoretical SLR excitation) revealed a significant difference (Figure S4a–c). Since our structures are covered with a highly reflective gold film, the resonances can be characterized by reflectance measurements. Meanwhile, bumps without film between the structures result in a low reflection response, as transmission through those regions increases. A direct comparison was performed using the extinction calculation (Figure S4c), eliminating transmittance and reflectance (100%-R-T). Although resonant peaks are observed in the bump without gold film arrays at similar positions, they are much less pronounced, indicating that a continuous gold film enhances coupling efficiency by involving SPP modes. Additionally, insight into the near-field side-view profiles of the primary *E*_pr_ resonances at φ = 0° was performed (Figure S4d,e). It revealed that the field is not confined to the individual structure and its sides but extends along the metal surface and penetrates into the thin gold film with a characteristic decay length. This observation proves SPP excitation in the metal layer, thereby confirming coupling via surface waves and HLPR origin.

Top-view simulations of the near-field at the periodic nanobumps provide deeper insight into plasmonic mode excitation. Figure 5 presents the spatial localization of plasmonic modes excited under s-polarization, where each column corresponds to a different azimuthal angle, and each row shows a different diffractional order of *E*_pr_ and *E*_se_. At φ = 0° (Figure 5a), where the plane of incidence (orange arrow) is aligned with the main grating axis, a strong field localization is observed at the edges of the nanobumps along the direction of electric field oscillation (yellow arrow). Both *E*_pr_ and *E*_se_ resonances exhibit similar field alignment along the main lattice, indicating that this plane is superior in that configuration. It is noteworthy that the *E*_se_ resonances at 799 and 678 nm exhibit lobes oriented at the corners, indicating diagonal 2D-type excitation. When the azimuthal angle is increased (Figure 5b,c), the near-field profiles exhibit a noticeable asymmetry and a rotation of lobes. This rotation reflects changes in the in-plane component of the wavevector that contribute to plasmonic excitation. At *E*_pr_ resonances, a stronger lobe is observed, with hotspot localization maintained along the main axis in PP, with a contribution from the rotated electric field at an angle. Meanwhile, at *E*_se_ resonances, a moderate angular electric-field contribution is also observed; in this case, the lobes are oriented, and the field is more concentrated along the diagonal axis. In both cases, there is a noticeable 15° and 30° azimuthal influence determining lobe orientation. Finally, at φ = 45° (Figure 5d), the incident beam is aligned with the diagonal grating direction, and the near-field hot spots across all resonances are distributed along the diagonal lattice axis, indicating efficient coupling into this grating configuration.

**Figure 5:**
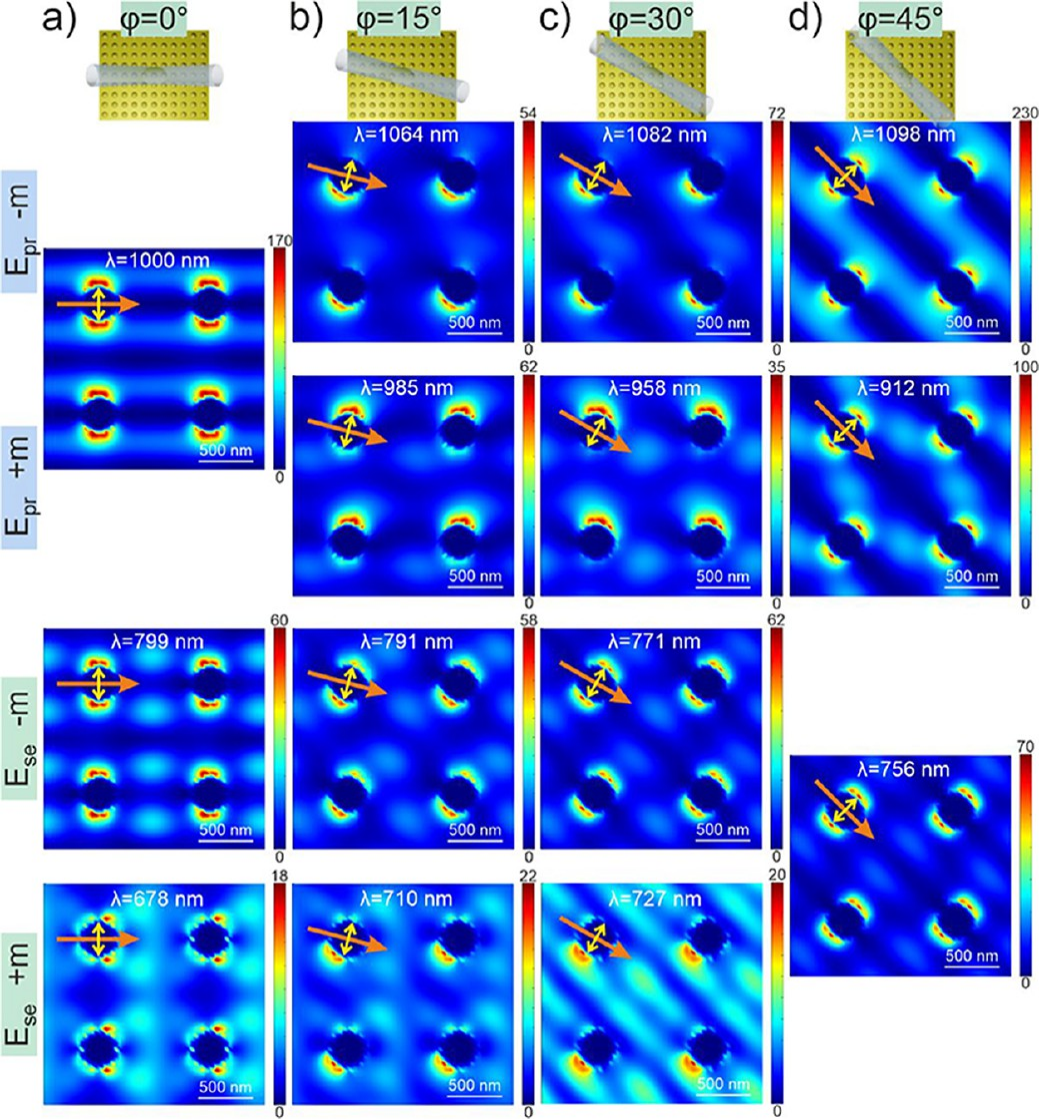
Top-view FDTD simulations of s-polarization near-field enhancement on gold nanobumps with a period of 1 μm at the resonant wavelengths for (a) φ = 0°, (b) φ = 15°, (c) φ = 30°, and (d) φ = 45° azimuthal angles. The first and second rows depict the primary *E*_pr_ resonances for negative and positive diffraction orders, respectively, while the third and fourth rows depict the secondary *E*_se_ resonances for negative and positive diffraction orders, respectively. Orange arrows indicate the light incidence direction, and yellow arrows denote the electric field oscillation direction. Graphs obtained at 87.5% of the structure’s height.

Comparing experimental peaks and their quality led to the conclusion that s-polarization resonant peaks at φ = 0° are narrower than those at φ = 45°. Meanwhile, the FDTD-calculated graph comparison in Figure 4a,d shows the opposite trend, with much narrower, deeper peaks at φ = 45°. Such a theoretical discrepancy can be explained by analyzing the top-view near-field enhancements of the *E*_pr_ peaks in the structures (Figure 5a,d). At φ = 0°, the coupling is governed by diffraction and plasmonic excitation along a principal lattice, leading to interaction between neighboring structures only in this axis, and coupling being defined by the lattice period. In contrast, at φ = 45° (Figure 5d), the excitation involves a 2D-type diffraction condition, where near-field maps reveal that the coupling is not based on direct longer-period (√2 μm) interaction. Instead, the stronger coupling in the diagonal is achieved through the interaction of shorter effective coupling pathways through the neighboring structures. Such shorter interaction length leads to smaller damping and more pronounced resonant peaks. However, the experimental gratings suffer from irregularities in the period, which are more significant in the diagonal lattice, thus leading to reduced coupling efficiency and shallower peaks.

The simulated reflectance spectra for p-polarization are depicted in Figure 6. Similar to s-polarization, the simulated resonances are slightly red-shifted relative to the experimental resonances. From all angles, the *E*_pr_ negative peak retains its depth and is the most intense resonance. Near-field simulations (insets of Figure 6) prove that this resonance (at ∼1180–1190 nm) is an out-of-plane mode with the field localization at the top of the structure. Unlike other resonances, this resonance depends strongly on the structure’s shape and height.^51^ The remaining spectral peaks are caused by the in-plane modes, characterized by the field localization at the lateral boundaries of the nanobump. While in the s-polarization case, the *E*_pr_ positive resonance manifests as two lobes at φ = 0° and φ = 45° angles. Under p-polarization, this resonant mode exhibits field localization only at a single side of the structure across all azimuthal angles, and the field is positioned at the side of the incident beam. This indicates the nonuniform excitation conditions of the positive and negative order plasmonic modes under p-polarization at zero azimuth. Meanwhile, at s-polarization, the excitation conditions are uniform since the field is perpendicular to the plane of incidence. Similar to the experimental results, near the attributed *E*_pr_ and *E*_se_ resonances, some additional peaks are visible (Figure 6b,c) that are due to coupling into several lattices excited with both polarizations (similar to Figure S3).

**Figure 6:**
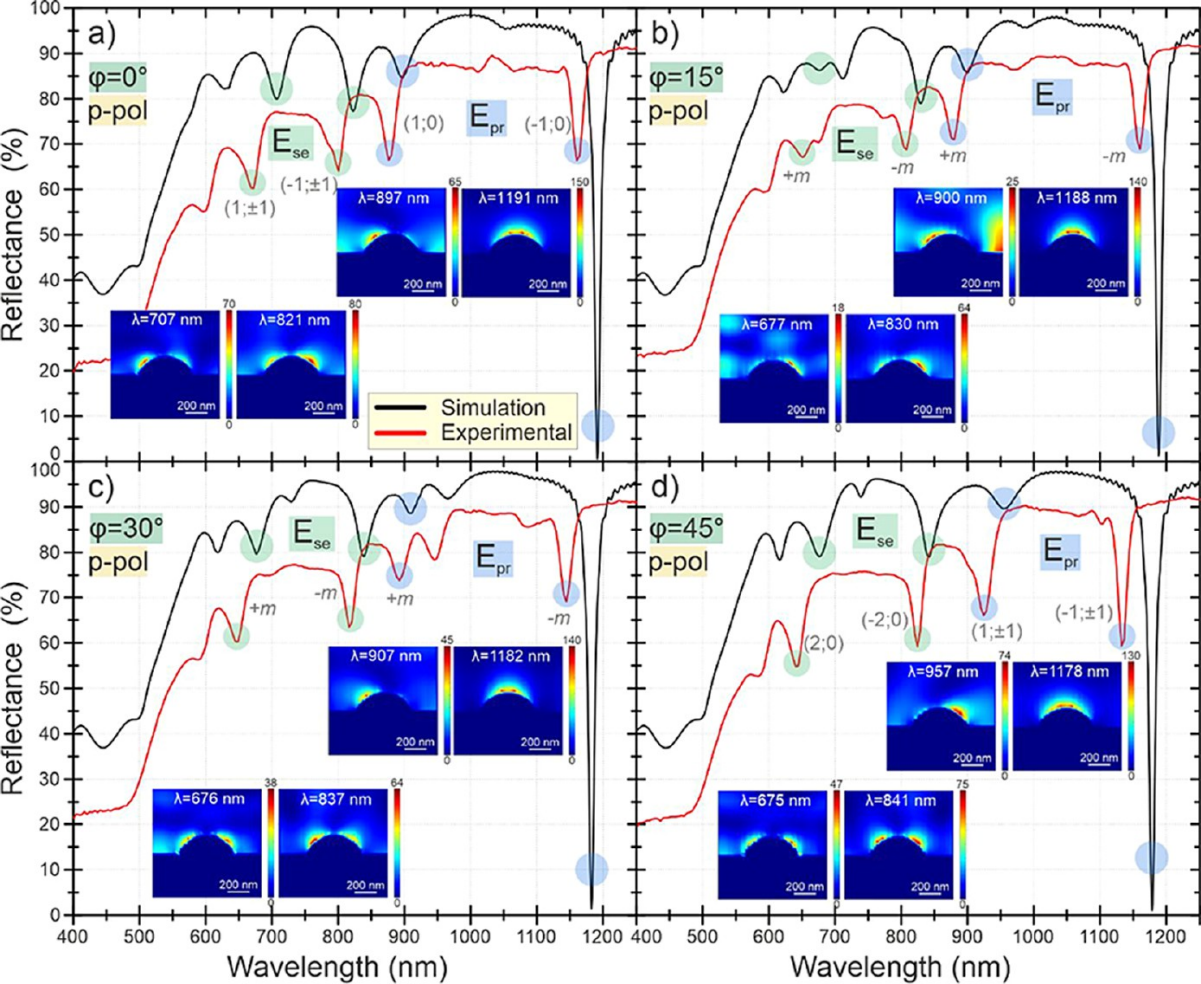
FDTD simulated (black line) and experimental (red line) reflectance spectra of p-polarized light response from gold nanobumps with a period of 1 μm at θ = 8°. The spectra are shown for azimuthal (a) φ = 0°, (b) φ = 15°, (c) φ = 30°, and (d) φ = 45°. Blue circles indicate the primary *E*_pr_ resonances, while green circles indicate the secondary *E*_se_ resonances. Insets show the side-view profiles of the simulated near-field enhancement on the structures at the corresponding resonant wavelengths.

The top-view graphs of the near-field at the resonant wavelengths under p-polarization are presented in Figure 7. The first row evidently illustrates the out-of-plane mode, with excitation located at the apex of the structure and a uniform field surrounding the bump across all angles. As in the s-polarization case, at φ = 0° (Figure 7a), the plane of incidence is aligned with the main grating axis, resulting in field localization along the sides of the nanobumps in this direction. In the case of p-polarization, the positive diffraction orders of both *E*_pr_ and *E*_se_ resonances experience enhanced field on the incidence side of the structure, whereas the negative order of the in-plane *E*_se_ mode is more pronounced on the propagation side. This single-sided field asymmetry at positive diffraction orders persists through all azimuthal rotations examined. The change in the φ angle further confirms the primary governing grating for the resonant modes. At φ = 15° and φ = 30°, the near-field of *E*_pr_ is maintained along the main grating axis, exhibiting a slight influence from the shifted excitation angle. Concurrently, the *E*_se_ undergoes a shift in respect to the azimuth, with the most significant enhancement occurring within the plane of incidence, while a portion of the field is oriented along the diagonal axis. When at φ = 45°, the diagonal plane becomes dominant (Figure 7d), the field becomes exclusively localized along the diagonal axis for all in-plane resonances.

**Figure 7:**
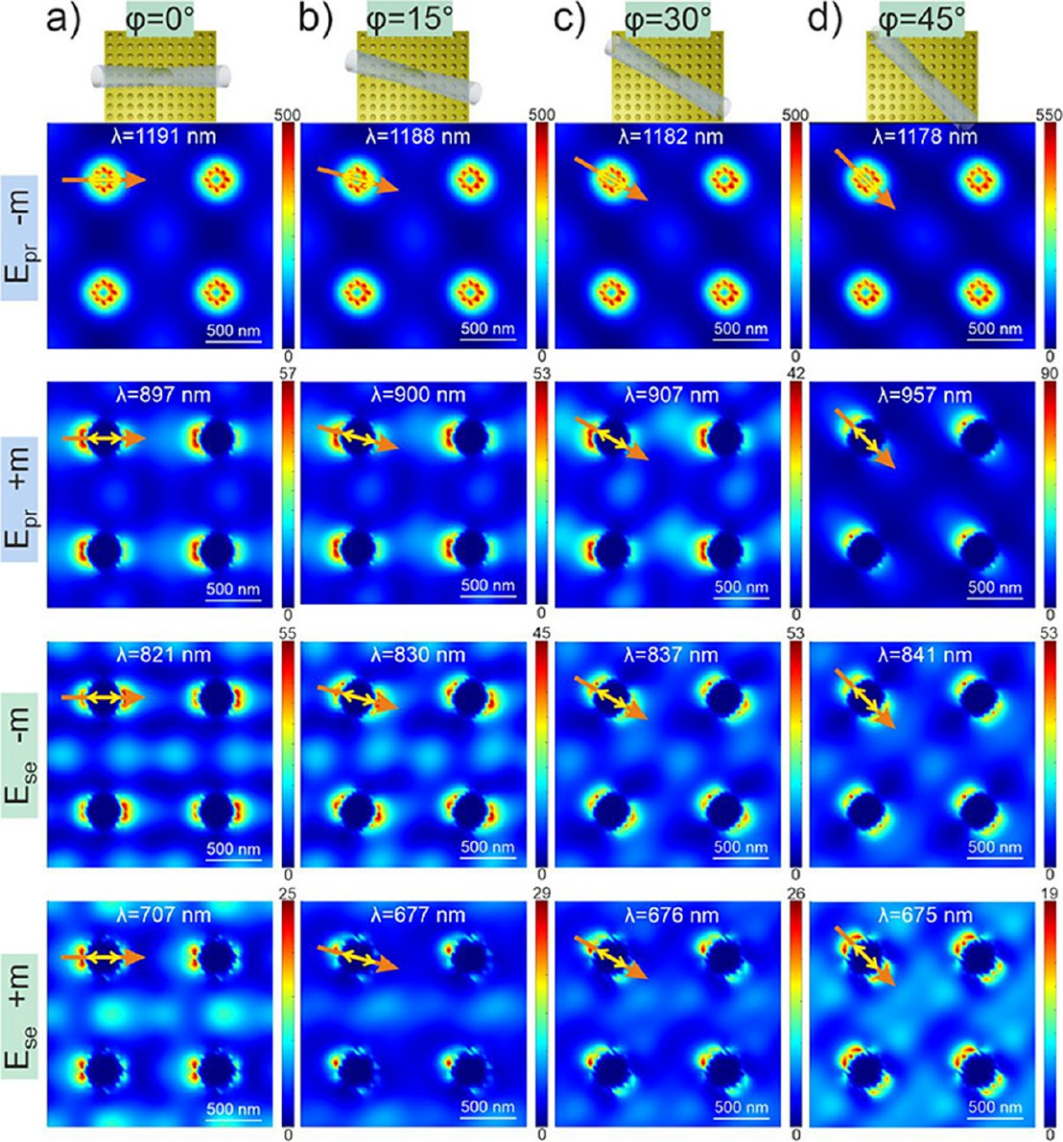
Top-view FDTD simulations of p-polarization near-field enhancement on gold nanobumps with a period of 1 μm at the resonant wavelengths for (a) φ = 0°, (b) φ = 15°, (c) φ = 30°, and (d) φ = 45° azimuthal angles. The first and second rows depict the primary *E*_pr_ resonances for negative and positive diffraction orders, respectively, while the third and fourth rows depict the secondary *E*_se_ resonances for negative and positive diffraction orders, respectively. Orange arrows indicate the light incidence direction, and yellow arrows denote the electric field oscillation direction. First row graphs were obtained at 100% height, while others—at 87.5% height of the structure.

The simulated Figure S4 graphs also give insight into the polarization mode origin. For the isolated bump array (without the gold film), which corresponds to a conventional SLR system, a weak resonance is observed only in s-polarization at 1000 nm, indicating lattice-diffraction-coupled LSPs. For bumps with a Au film, the resonance persists at a similar position but with higher intensity, suggesting that this mode is SLR-mediated and greatly enhanced by interactions with SPPs in the metal film. Meanwhile, for p-polarization, strong resonances appear only in the presence of the gold film, indicating that the mode origin is more closely related to the grating-excited SPPs when the electric field component is normal to the metal surface.

Summarizing, a more pronounced directional asymmetry is observed under p-polarization excitation, originating from the electric field vector and its coupling to the SPP modes. Given the excitation’s oblique angle and the electric field’s component parallel to the plane of incidence, the diffraction-based excitation of the SPPs modifies the wavevector in opposite ways for positive and negative orders. This imbalance in momentum matching manifests as a single lobe in the near-field for p-polarization resonances. Conversely, under s-polarization, the electric field is perpendicular to the incidence plane, resulting in a symmetric coupling to both propagation directions.

One of the advantages of the DLW method is the flexibility in fabricating freely chosen lattice geometries. For further azimuthal grating-coupling exploration, bump gratings with rectangular symmetry were fabricated by reducing the in-line period along the scanning direction (0.865 and 0.75 μm), while maintaining a constant 1 μm perpendicular periodicity. The comparison of the reflectance spectra is given in Figure S5. The square lattice (Figure S5a) exhibits smooth, symmetric resonance shifts with azimuthal angle (marked with dashed lines), with similar responses at φ = 0° and φ = 90° due to the continuous coupling transition from the principal 1 μm-period lattice to the diagonal lattice and back to the 1 μm-lattice. Since the in-plane component of the incident wavevector is perfectly aligned with either principal or diagonal lattices at the azimuthal angles of φ = 0/90° and φ = 45° (Figure S5a­(iii)), the most efficient coupling and thus the sharpest resonant peaks are observed at these high-symmetry angles, with the reduced coupling efficiency at intermediate angles.

In contrast, the rectangular arrays (Figure S5b,c) exhibit more complex and fragmented spectral behavior due to symmetry breaking and changes in diffraction-mode excitation conditions along the two principal lattice directions. Such anisotropic excitation conditions affect not only the mismatch in resonant wavelengths at φ = 0° and φ = 90° but also the coupling to different lattices at intermediate azimuthal angles. At 0.865 μm in-line period, the additional effective coupling to oblique lattices is achieved at φ = 30° (lattice vector of (2,1)) and φ = 60° (lattice vector of (2,3)). This makes the resonant peaks at these angles more pronounced than at other intermediate angles. In contrast, at 0.75 μm in-line period, the effective diffraction-based coupling is achieved at φ = 15° (lattice vector of (5,1)) and φ = 45° (lattice vector of (4,3)). In such a rectangular alignment, the resonance shift does not follow a continuous trend but instead exhibits a nonuniform, fragmented shift, with discontinuities (around φ = 45°) corresponding to transitions in coupling between the perpendicular principal lattices. A further reduction of the in-line period leads to the formation of anisotropic gratings that approach quasi-1D and 1D behavior, in which isolated resonant peaks dominate.^72^ These results show that while rectangular lattices provide increased flexibility and access to a richer k-space, they also introduce complexity in resonance behavior due to anisotropic coupling conditions. Therefore, the following research will focus solely on the symmetric grating, which offers more intuitive and more easily defined resonance control.

### 2.3 Double-Angular Control of Resonant Wavelengths

To further explore the angular dependence of diffractively coupled plasmonic excitation, the azimuthal rotation study was extended to different angles of incidence. This approach enables double-angular manipulation of the resonances. The sample was measured at incident angles θ = 8°, 15°, and 30°, at the same φ as previously, with a 15° step. Figure 8 presents the experimentally determined dependence of wavelength on azimuthal angle, accounting for the angle of incidence. The measured reflectance spectra at θ = 15° and 30°, with resonances attributed to primary or secondary, are shown in Figure S6. Since θ impacts the diffraction condition, the plasmonic excitation highly depends on this parameter as well. An increase in the angle leads to an increasing separation of the diffractively coupled resonances at PI and DP (p-polarization and s-polarization), and a redshift of the single resonance at PP (s-polarization). Angular shift leads to a redshift of negative and a blueshift of positive order of diffraction resonance in p-polarization (Figure 8b,d). Meanwhile, the s-polarization (Figure 8a,c) response depends on the azimuthal angle: a blueshift of resonances is observed at azimuthal orientations close to the 1D-type excitation in PP (φ = 0°/90° and 15°/75° for the *E*_pr_, and φ = 45° and 30°/60° for the *E*_se_). At other φ angles, the same tendency as in p-polarization is maintained, with a redshift of negative order resonance. This indicates that polarization also plays an important role in determining the diffraction-induced plasmonic excitation, and which angular shift, azimuthal or excitational, has a greater effect on the resonant spectral position. It has been demonstrated that a larger incident angle yields steeper dependence curves, indicating more sensitive resonance control with an azimuthal shift. The overall spectral dispersion with incident angle is weaker for the secondary modes (Figure 8c,d). At higher incidence angles (Figure S6d), the separation of the resonant modes becomes pronounced, shifting the positive diffraction order into the metal absorption zone, where it is no longer observable. Meanwhile, additional unassigned resonances (Figure S6b,d) emerge in the spectra. These originate from higher-order diffractions that redshift out of the gold absorption zone as the incident angle increases. Figure 8 insets of the polar plots demonstrate that peak shifts exhibit a similar tendency across all incident angles, emphasizing the rotational symmetry of the resonances. Such double-angle control enables continuous tuning of the plasmonic excitation conditions without altering the grating geometry.

**Figure 8:**
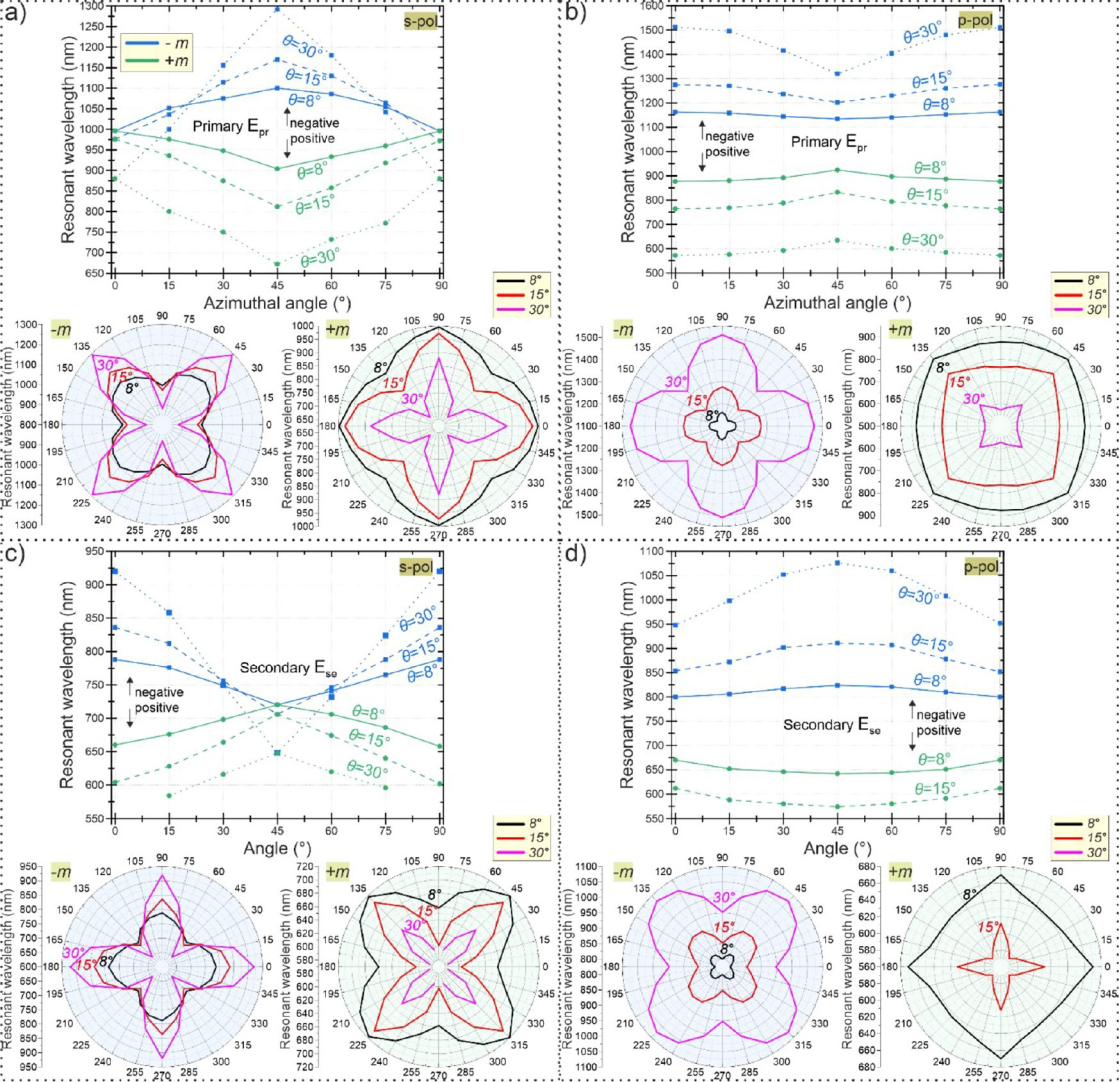
Experimental (a,b) primary *E*_pr_ and (c,d) secondary *E*_se_ resonant wavelength dependence on azimuthal angle, with incident angle influence for (a,c) s- and (b,d) p-polarization. Blue lines mark the positive-order diffraction resonances, and green lines the negative-order resonances, at incident angles of 8° (solid lines), 15° (dashed lines), and 30° (dotted lines). The insets show polar plots of the same resonant peaks, with negative-order displayed in a blue background and positive-order in a green background.

By implementing eqs 3–5, the tendency for resonant wavelength dependence on the incidence angle was calculated and presented in Figure S7. The results are depicted for the special case of 1D diffraction (Figure S7a), calculated using [Disp-formula eq4]) and ([Disp-formula eq5]) formulas, as well as for the 2D diffraction (Figure S7b). Marked symbols, representing experimental values, are in consistent agreement with the curves at all three angles of incidence: 8°, 15°, and 30°. For diagonal 2D-type diffraction excitation, s-polarization results exhibit better agreement with the calculations than p-polarization data, which can be attributed to the purely in-plane component in s-polarization.

Finally, the qualitative assessment of the resonances has been performed by evaluating the modified quality factor of each peak. The tendency for azimuthal rotation with respect to the incidence angle and polarization is provided in Figure 9. Unlike the conventional Q-factor, the MQ-factor, as described in eq 6, in this case, provides a more reliable basis for comparing resonances across all azimuthal orientations. Narrow but shallow resonances, obtained at φ = 15° and φ = 30°, may artificially increase and distort the Q-factor tendency, whereas the MQ-factor simultaneously accounts for line width and depth, yielding a physically meaningful measure for comparison. Under s-polarization, the resonance quality is generally poorer than under p-polarization, as reflected in lower MQ-factor values. The dominant are primary *E*_pr_ resonances with higher qualities, while *E*_se_ peaks remain comparatively weaker. All resonances exhibit a pronounced maximum at azimuthal angles aligned with the main and diagonal lattice axes (φ = 0° and 45°). Such a methodology, incorporating azimuthal rotation and quality measurements, could be used to experimentally determine the dominant diffraction axes of the fabricated grating. Under s-polarization, for θ = 8° (Figure 9a), the highest MQ values occur at φ = 0°, followed by a sharp decrease at intermediate angles, indicating that these resonances are tightly confined to the excitation along the main symmetry direction of the grating. A moderate secondary maximum at φ = 45° corresponds to coupling into the diagonal lattice, although with lower efficiency. Increasing the incidence angle to 15° and 30° (Figure 9b,c) enhances the angular selectivity of the peaks, maintaining similar quality along the main grating and reducing the quality of the diagonal-coupled resonances. At all incident angles, consistent with the tendencies observed in Tables 1 and 2, the s-polarization resonances exhibit higher quality at φ = 0° than at φ = 90°, showing stronger coupling when the plane of incidence is parallel, and the electric field is perpendicular to the scanning lines.

**Figure 9:**
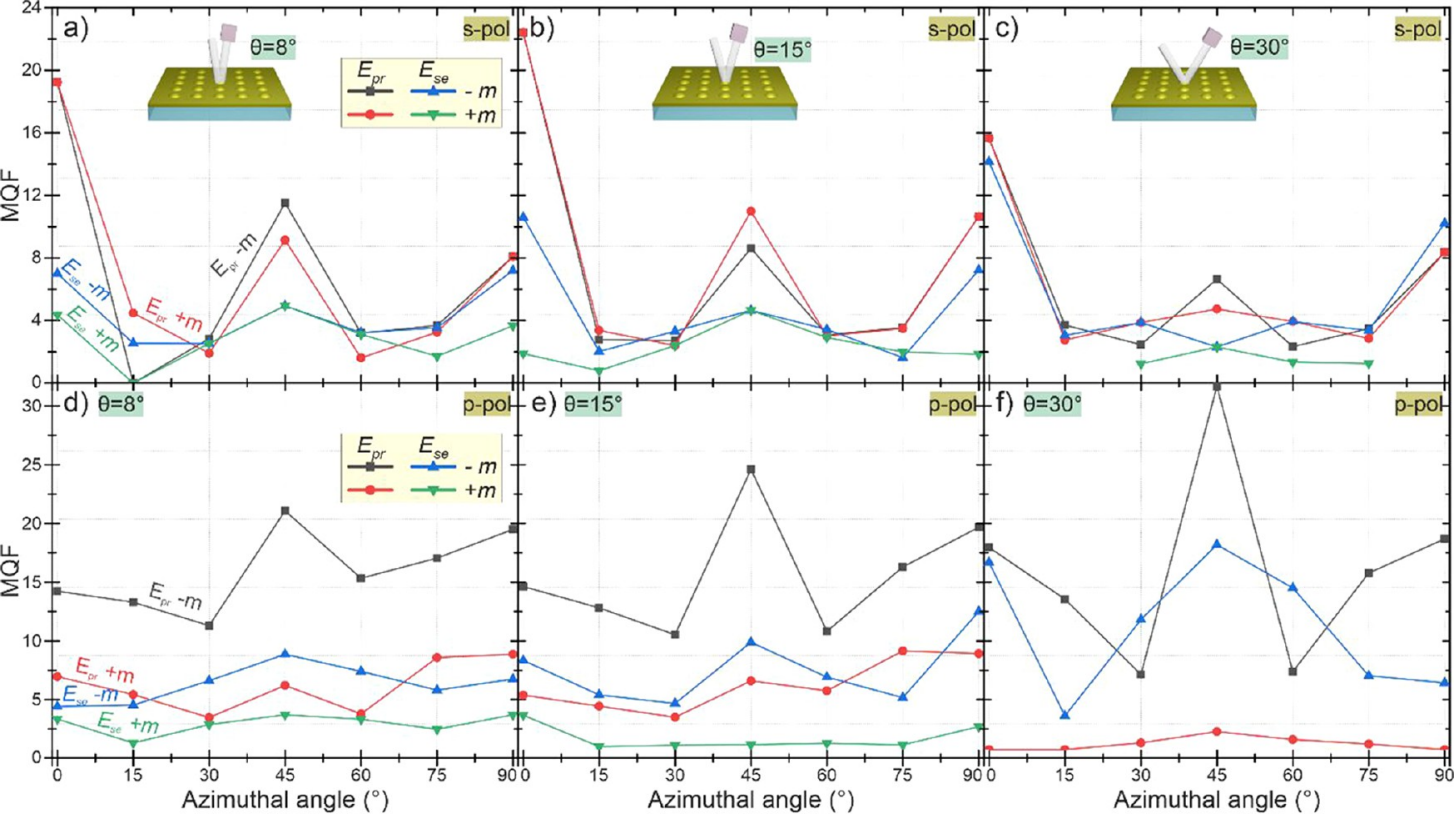
Modified quality factor dependence on the azimuthal angle for the experimental resonances obtained under (a–c) s-polarization and (d–f) p-polarization at (a,d) 8°, (d,e) 15°, and (c,f) 30° incident angles. Black lines denote *E*_pr_ negative, red lines *E*_pr_ positive, blue lines *E*_se_ negative, and green lines *E*_se_ positive resonances.

Using p-polarization, the out-of-plane mode (primary negative *E*_pr,_ −*m*) dominates across all azimuthal angles, with a particularly large MQ-factor. At all investigated angles of incidence, the quality is higher at the φ = 90° configuration (MQ around 20) compared to φ = 0° (MQ around 15), indicating that p-polarization plasmonic excitation is more efficient when the plane of incidence and the electric field are perpendicular to the scanning lines. However, the highest-quality resonances are achieved at φ = 45° angles, demonstrating that coupling to a diagonal grating provides the most favorable conditions. Increasing the incidence angle improves the peak quality, from MQ = 20 at 8° to MQ = 32 at 30°. Other resonances, though weaker, follow a similar azimuthal tendency. Overall, a general trend emerges: the highest-quality peaks are obtained at excitation angles aligned with either the main or the azimuthal lattice direction, with a sharp drop in quality at intermediate angles. This behavior reflects the symmetry of the 2D grating and a directional dependence of the diffraction-coupled resonances.

## 3 Conclusions

In this paper, we have insightfully studied the diffraction-based excitation of the hybrid plasmonic resonances in a 2D periodic nanobump grating. The resonances, arising from the hybridization of grating-induced surface plasmon polaritons, localized surface plasmons, and diffracted light at the lattice, were identified and assigned to their origins. The experimentally observed spectral features are well described by hybrid lattice plasmon resonances, whose behavior is governed by coupling to the array’s main or diagonal axes. The results were fully supported by combining angle-resolved reflection measurements with theoretical grating-diffraction formulas, FDTD simulations, and near-field mappings. The azimuthal-angle study revealed strong directional selectivity in the modes, thereby providing additional spectral tunability of the resonant peaks. The wavelength positions systematically shift with azimuthal variation due to the varying in-plane components of the diffracted wavevectors. Near-field simulations demonstrated that s-polarization generates more symmetric in-plane field distributions, whereas under p-polarization, asymmetric single-lobe patterns are obtained due to oblique excitation. A thorough analysis of the modified quality factor indicated that the high-quality resonances are obtained when the excitation is aligned with the high-symmetry directions of the lattice – either along the main grating (φ = 0°, 90°) or along the diagonal axes (φ = 45°). Finally, the novelty of this work lies in the demonstration and investigation of dual-angular control of both azimuthal and incidence angles, further expanding the resonance control region across the entire Vis-NIR range. Overall, the combined experimental-theoretical approach provides a comprehensive understanding of the excitation and tunability of diffraction-based plasmonic resonances in symmetric 2D arrays.

## 4 Experimental Section

### 4.1 Materials

A 100 nm-thick gold film was deposited on 1 mm-thick soda-lime optical glass using a magnetron sputter coater (Quorum Q150T) at a deposition rate of ∼0.26 nm/s.

### 4.2 Nanostructures Array Formation

Gold nanostructures were fabricated on the thin gold film using direct laser writing (DLW) using third-harmonic (343 nm) of 300 fs laser pulses generated by a Yb:KGW-based fs-laser (Pharos, Light Conversion). Structures were fabricated using a tightly focused (∼800 nm) femtosecond laser beam with 0.8 nJ pulse energy to produce the hemispherical bumps on the surface. The structures were produced by scanning a laser pulse along a line at a specified speed and frequency, so that individual pulses could be distinguished by the required period. The period along the perpendicular axis was determined by the spacing between the scanning lines. The period of the uniform 2D grating was chosen to be 1 μm. The size of the produced symmetrical 2D array was 3 × 3 mm^2^.

### 4.3 Characterization

The periodic array was characterized using a spectrophotometer (Photon RT, Essentoptics) and a scanning electron microscope (Helios NanoLab650). The reflectance spectra in the wavelength range from 0.4 to 1.6 μm were measured at different light polarizations (s- and p-polarization) at several incidence angles θ (8°, 15°, 30°). The azimuthal angle φ was varied from 0° to 180° in 15° increments by manually rotating the sample on the angular scale. Angle φ = 0° corresponds to the case when the plane of incidence is parallel to the laser scanning lines, and at φ = 90°, the plane of incidence is perpendicular to the scanning lines. The spot size of the impinging light was a 2 mm diameter circle. SEM characterization of the bump array was performed after reflectance spectrum measurements.

### 4.4 Theoretical Calculations

Resonant wavelengths for plasmonic excitations were computed using the 2D diffraction grating formula, with gold dispersion taken from Johnson and Christy.

### 4.5 Numerical Modeling

Numerical simulations were performed using the Lumerical FDTD solver. A single unit cell of the 2D periodic gold-bump grating (period Δ*x* = 1 μm) was modeled in 3D using Bloch boundary conditions along the periodic axis and PML in the propagation direction. The substrate (*n* = 1.51 at 900 nm), the 100 nm gold layer, and the bump geometry (from SEM measurements) were defined as in the experiment, with the bump interior assigned *n* = 1. Gold dispersion was taken from Johnson and Christy. Oblique illumination was implemented using BFAST, with a broadband source from 400 to 1600 nm and 1 nm spectral resolution. A uniform mesh was used, obtained by discretizing the unit-cell period into 32 points (mesh size *d*_*x*_ = Δ*x*/32). A time-step multiplier of 0.9 was applied to avoid instabilities arising from dispersive materials under oblique incidence. Simulations were performed for the same incidence angles as in the experiment (θ = 8°, 15°, 30°) and for both s- and p-polarizations. Due to symmetry, azimuthal angles were simulated only from 0° to 45° in 15° steps.

0th-order reflection spectra were obtained using 2D frequency-domain FDTD power monitors positioned above the gold structure. Electric-field distributions were computed using a full 3D monitor covering the entire unit cell, enabling the evaluation of in-plane fields at different heights and vertical oblique field cuts to visualize resonance profiles to extract a field cross-section at an arbitrary angle θ from a 2D field matrix. The coordinate system is recentered so that the origin coincides with the center of the matrix.

For a matrix *A*(*i*, *j*, *k*) of size *N*_*x*_ × *N*_*y*_ × *N*_*z*_, the centered coordinates are defined as

$$i_{0}=\frac{N_{x}-1}{2},\;j_{0}=\frac{N_{y}-1}{2}$$

A linear cut at an angle θ is parametrized by the line passing through the origin,

$$i(r)=\frac{r}{d_{x}}\cos\;\theta -i_{0},\;j(r)=\frac{r}{d_{x}}\sin\theta -j_{0}$$

where $r\in [-d_{x}\sqrt{{i_{0}}^{2}+{j_{0}}^{2}},d_{x}\sqrt{{i_{0}}^{2}+{j_{0}}^{2}}]$ , with step of *d*_*x*_, the field value along the cut is obtained from *A*(*i*, *j*, *k*) using nearest-neighbor or bilinear interpolation for noninteger indices. Within the limits of the matrix, this procedure yields a one-dimensional field profile corresponding to a slice through the structure at the specified angle; here, the plane is

$$E(r,z)=A\left(\frac{r}{d_{x}}\cos\;\theta -i_{0},\frac{r}{d_{x}}\sin\theta -j_{0},k\right)$$

Each reflectance spectrum simulation required ∼2 min per angle, while near-field calculations at resonance wavelengths required ∼14 min per resonant wavelength.

## Supplementary Material



## Notes

Reflectance spectra comparison of DP resonance excitation with both polarizations; main and diagonal lattices resonance attribution; resonance at intermediate azimuthal angles comparison; reflectance spectra azimuthal shift for higher AOIs; and theoretically calculated resonance dependence on AOI (PDF)
