# Supplementary material for: Grating-Coupled Plasmonic Resonances in Symmetric 2D Gold Nanobump Grating: Theory Meets Experiments
Source: ACS Appl Mater Interfaces. 2026 May 19;18(23):33007–23. doi: 10.1021/acsami.6c04043 (PMC13288391; doi:10.1021/acsami.6c04043)
Supplement: Supplementary file 1 [file am6c04043_si_001.pdf]

Supporting Information

Grating-Coupled Plasmonic Resonances in Symmetric 2D Gold Nanobump Grating: Theory Meets Experiments

Kernius Vilkevičius,[*,†] *Lucciano A. Letelier,*[‡] *Lina Grinevičiūtė,*[‡] *and Evaldas Stankevičius*[*,†]

[†] Plasmonics and Nanophotonics Laboratory, Department of Laser Technologies, Center for Physical Sciences and Technology (FTMC), Savanoriu Ave. 231, LT-02300, Vilnius, Lithuania

[‡] Optical Coatings Laboratory, Department of Laser Technologies, Center for Physical Sciences and Technology (FTMC), Savanoriu Ave. 231, LT-02300, Vilnius, Lithuania

Corresponding authors' email: kernius.vilkevicius@ftmc.lt; evaldas.stankevicius@ftmc.lt

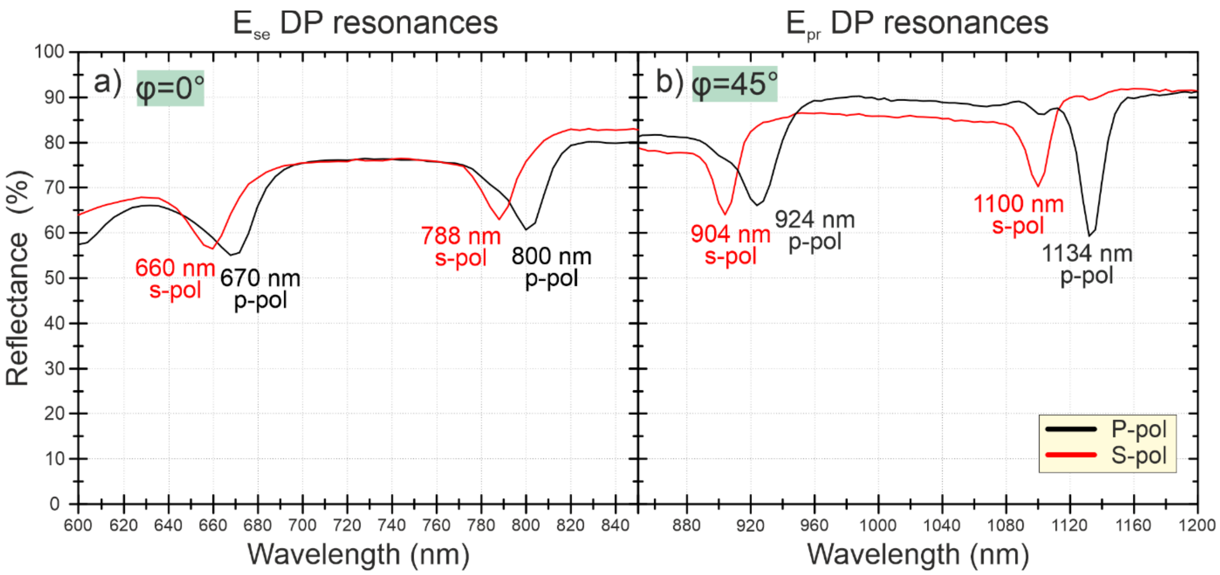

Figure S1. Reflectance spectra of 2D-origin diffraction (a) secondary resonances at φ = 0° and (b) primary resonances at φ = 45°, both being excited in the diagonal plane. The black line marks the spectra for p-polarized light and the red line for s-polarized light.

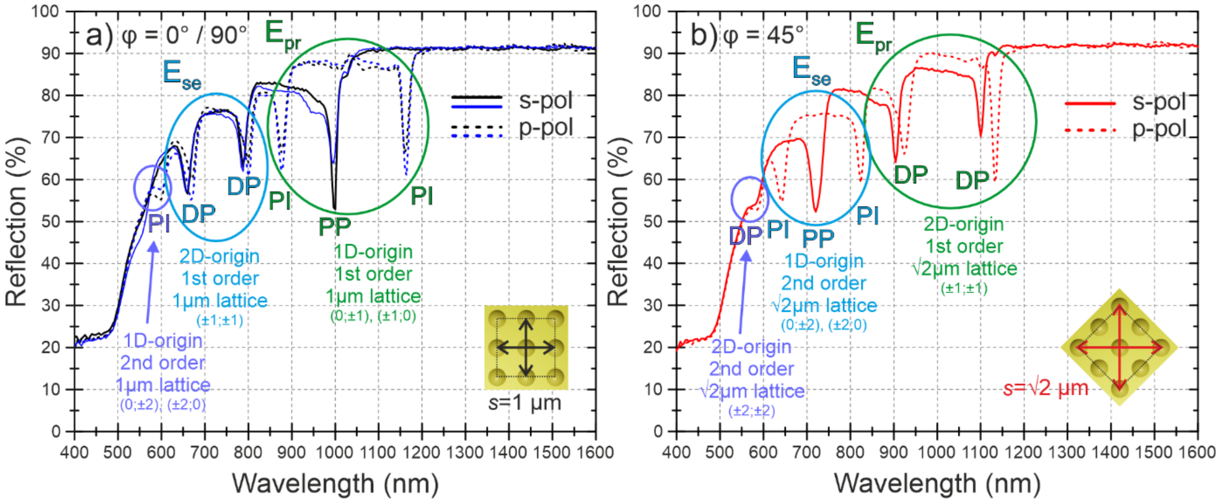

Figure S2. Reflectance spectra of (a) main 1 µm lattice resonances at φ = 0° / 90° and (b) diagonal √2 µm lattice resonances at φ = 45°, with attributed origins of the peaks. Solid lines mark s-polarization response, while dashed lines are for p-polarization.

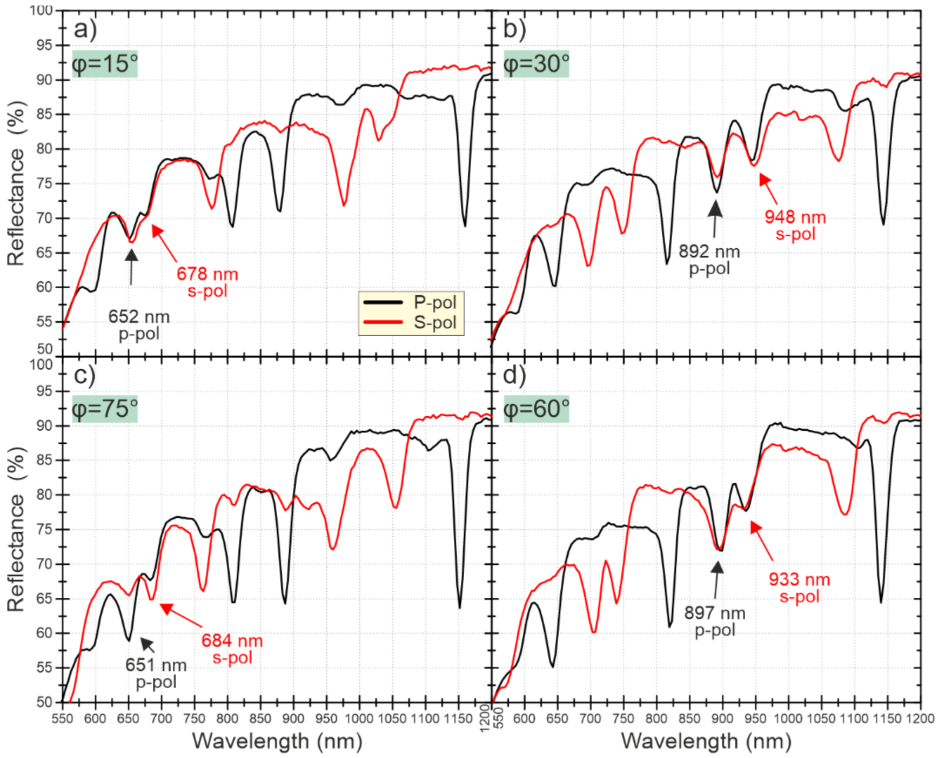

Figure S3. Comparison of the resonances, excited in both s- (red line) and p-polarizations (black line) at (a) φ = 15°, (b) φ = 30°, (c) φ = 75°, and (d) φ = 60° azimuthal angles. The response is shown for an angle of incidence of 8°.

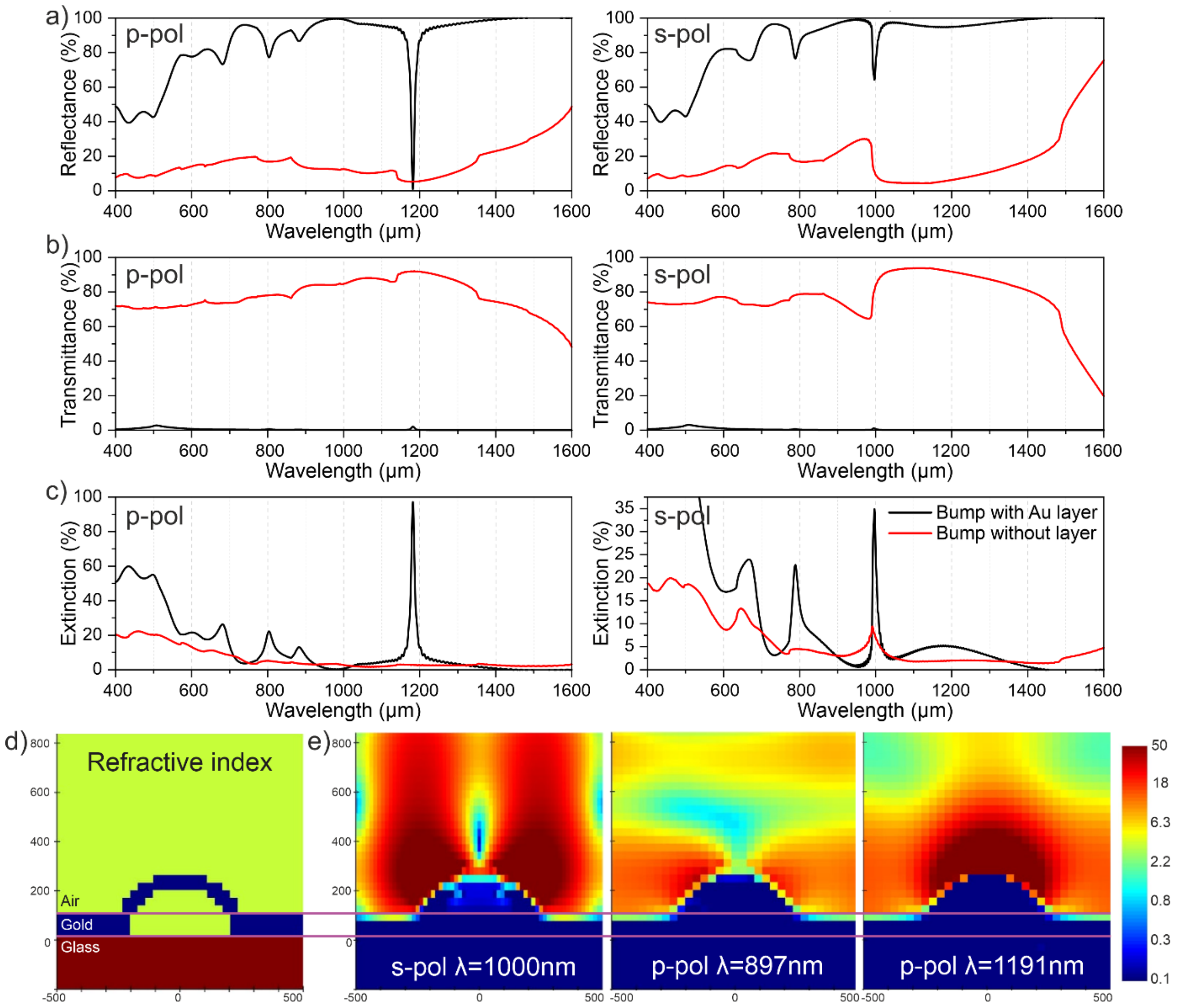

Figure S4. Simulated (a) reflectance, (b) transmittance, and calculated (c) extinction spectra of periodic bumps with gold film (black line) and isolated bumps without gold film (red line). The results are simulated at $\theta = 8°$ and $\varphi = 0°$. (d) Refractive index distribution in the simulated structure using FDTD, and (e) near-field enhancement of the s-polarization response at 1000 nm and p-polarization response at 897 nm and 1191 nm resonances. The violet line marks the Au film-air and film-glass interfaces.

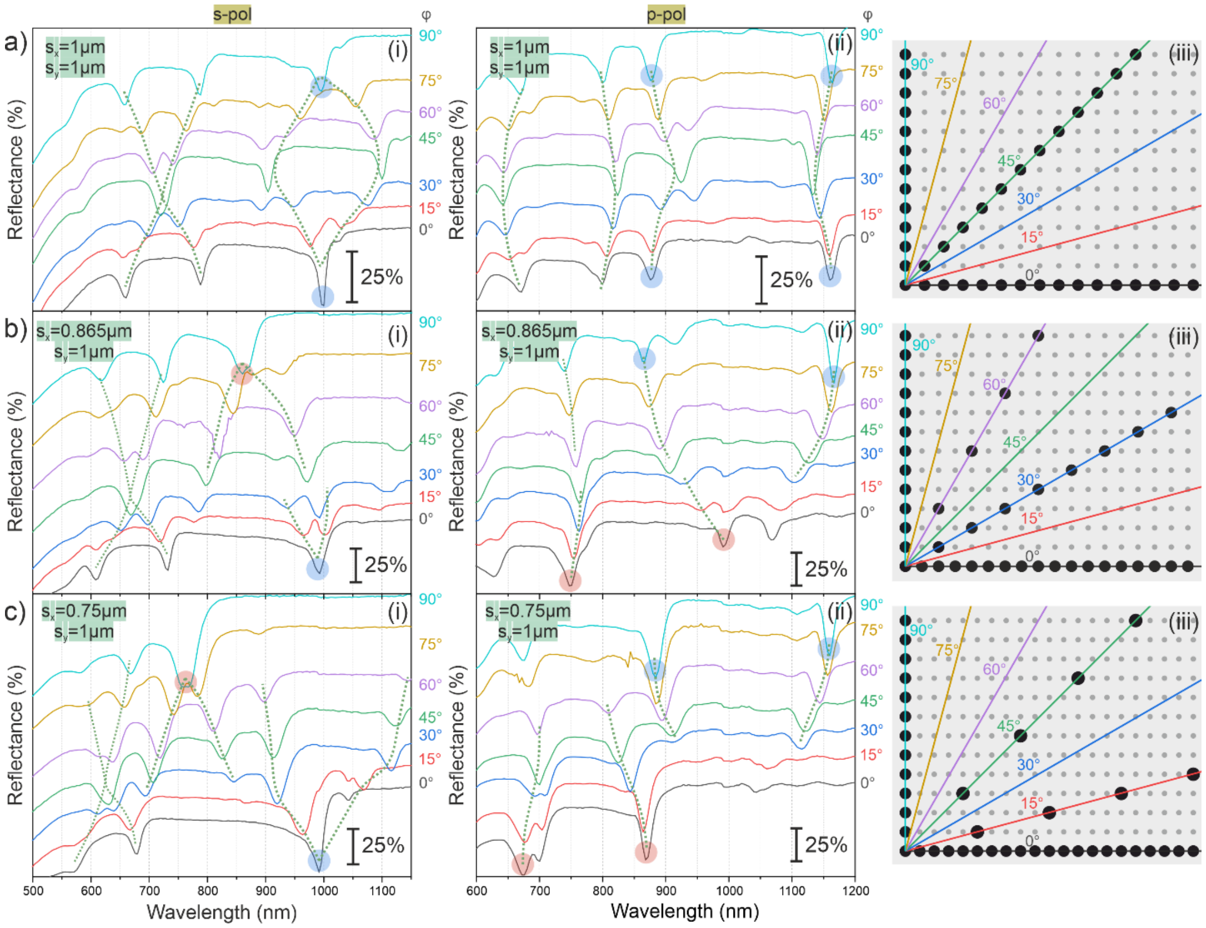

Figure S5. Reflectance spectra dependence on azimuthal angle of (a) a squared 1 μm period array, (b) a rectangular with 0.865 μm in-line period array, and (c) a rectangular with 0.75 μm in-line period array. The reflectance is for s-polarization (i) and p-polarization (ii), with blue circles marking resonances of a larger-period (1 μm) lattice and red circles marking resonances of a smaller-period lattice. Dashed lines mark the resonance shift. Azimuthal rotation coupling into different lattices of the gratings (iii), where black dots mark the aligned lattice.

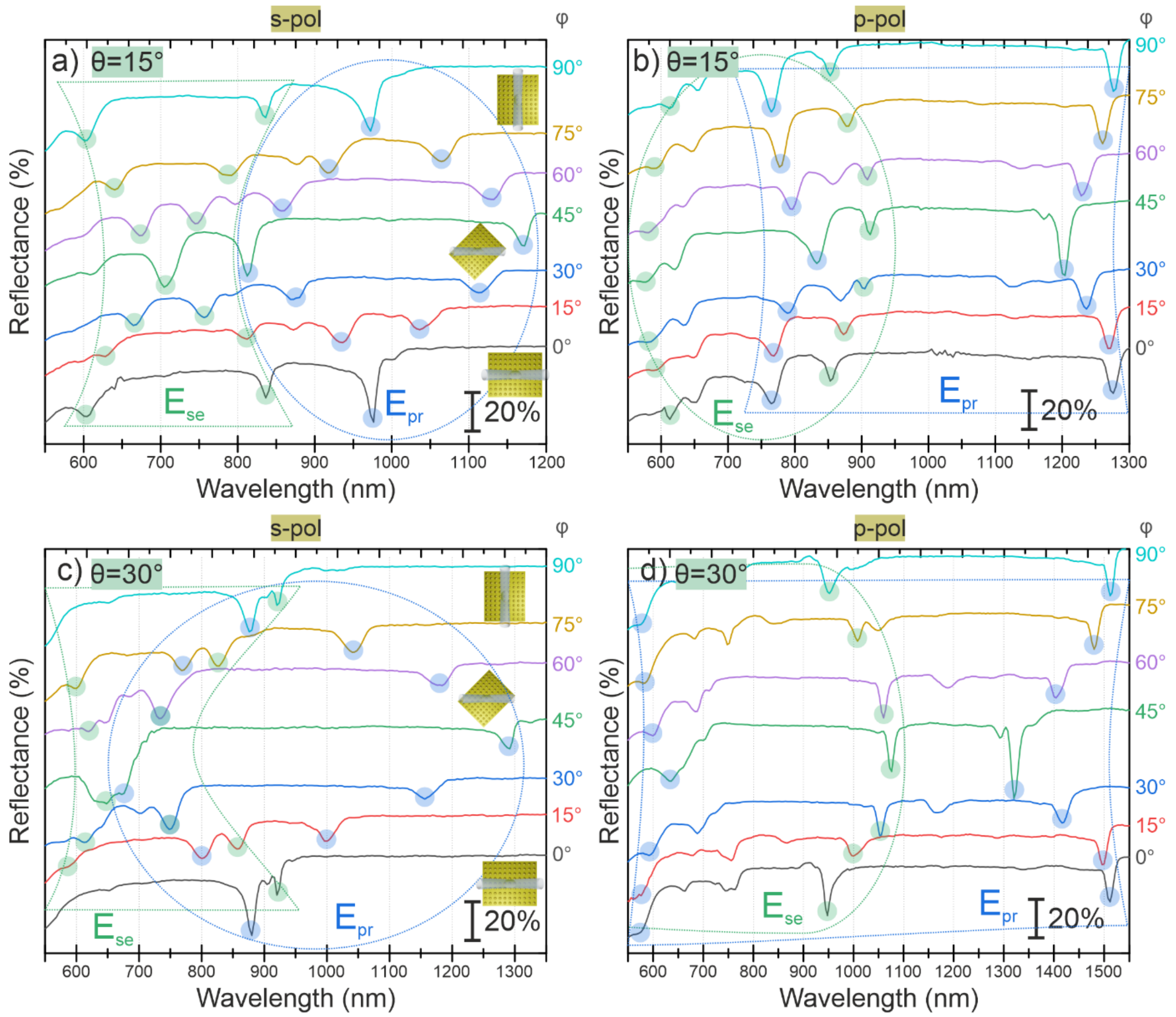

Figure S6. Reflectance spectra of gold nanobumps grating with a period of 1 μm at different azimuthal angles from φ = 0° to φ = 90° with a step of 15° for (a,c) s- and (b,d) p-polarization at (a,b) θ = 15° and (c,d) θ = 30° angles of incidence. Blue circles mark the primary E_{pr} resonances, while green ones mark secondary E_{se} resonances.

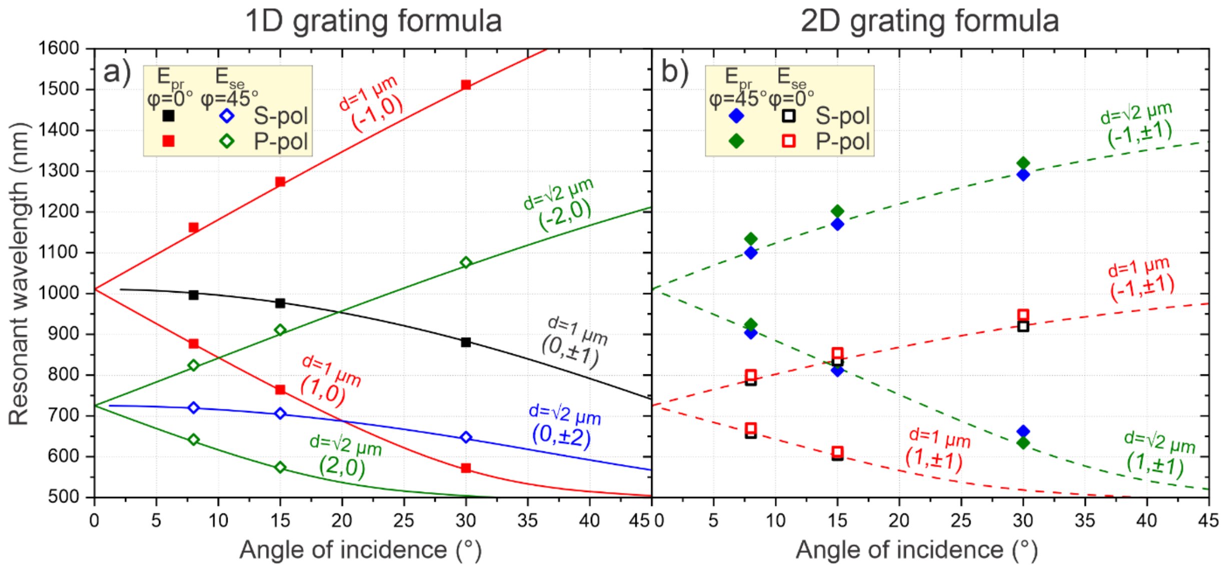

Figure S7. Theoretically calculated wavelength dependence on the incident angle using (a) 1D and (b) 2D diffraction formula. Curves are calculated for the main 1 μm grating at φ = 0° (black, red) and the diagonal √2 μm grating at φ = 45° (blue, green). Experimentally measured primary E_{pr} (solid symbols) and secondary E_{se} (empty symbols) resonance values are marked in squares (φ = 0°) and diamonds (φ = 45°) for s- (black, blue) and p-polarization (red and green).
